# 4D Force Detection of Cell Adhesion and Contractility

**DOI:** 10.1021/acs.nanolett.2c03733

**Published:** 2023-03-28

**Authors:** Nafsika Chala, Xinyu Zhang, Tomaso Zambelli, Ziyi Zhang, Teseo Schneider, Daniele Panozzo, Dimos Poulikakos, Aldo Ferrari

**Affiliations:** † Laboratory of Thermodynamics in Emerging Technologies, Department of Mechanical and Process Engineering, ETH Zurich, Sonneggstrasse 3, 8092 Zurich, Switzerland; ‡ Laboratory of Biosensors and Bioelectronics, Institute for Biomedical Engineering, ETH Zurich, Gloriastrasse 35, Zurich 8092, Switzerland; § Courant Institute of Mathematical Sciences, New York University, 5th Avenue 60, New York, New York 10011, United States; ∥ Department of Computer Science, University of Victoria, 3800 Finnerty Road, Engineering & Computer Science Building, Victoria, BC V8P 5C2, Canada; ⊥ Experimental Continuum Mechanics, EMPA, Swiss Federal Laboratories for Material Science and Technologies, Überlandstrasse 129, 8600 Dübendorf, Switzerland; # Institute for Mechanical Systems, Department of Mechanical and Process Engineering, ETH Zurich, Leonhardstrasse 21, 8092 Zürich, Switzerland

**Keywords:** traction force microscopy, FluidFM, single-cell force spectroscopy, cell compression, actomyosin contractility

## Abstract

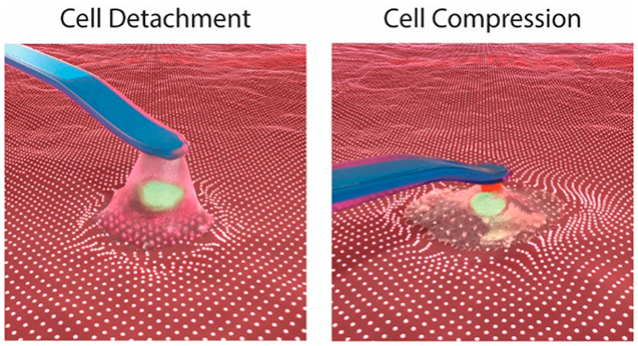

Mechanical signals establish two-way communication between mammalian cells and their environment. Cells contacting a surface exert forces via contractility and transmit them at the areas of focal adhesions. External stimuli, such as compressive and pulling forces, typically affect the adhesion-free cell surface. Here, we demonstrate the collaborative employment of Fluidic Force Microscopy and confocal Traction Force Microscopy supported by the Cellogram solver to enable a powerful integrated force probing approach, where controlled vertical forces are applied to the free surface of individual cells, while the concomitant deformations are used to map their transmission to the substrate. Force transmission across human cells is measured with unprecedented temporal and spatial resolution, enabling the investigation of the cellular mechanisms involved in the adaptation, or maladaptation, to external mechanical stimuli. Altogether, the system enables facile and precise force interrogation of individual cells, with the capacity to perform population-based analysis.

The life and function of mesenchymal cells require their anchoring to the extracellular environment,^1^ which is sustained along the entire cell cycle.^2^ Attachment to the local substrate is mediated by specialized transmembrane receptors of the integrin family, which form molecular complexes with intracellular proteins, the focal adhesions (FAs^3^). Integrin binding to extracellular matrix (ECM) ligands precedes and enables the assembly of mature FAs. It ensures physical anchorage,^4^ which passively resists detachment by external forces, maintaining the cell docking until the establishment of full adhesion.^5^

When a sufficient number^6^ and density^7^ of integrin receptors are engaged, the FA matures eventually clutching with the actin cytoskeleton.^8^ This connection enables the exertion of contractile forces generated by actomyosin and their transmission to the ECM to actively deform and remodel the soft extracellular environment.^9,10^ In addition, it contributes to further actin recruitment at the adhesion site^11^ in a dynamic interplay that governs cellular activities, from migration to organogenesis.^12^

Precise measurements of the force interplay between the cell and its surroundings are key to the understanding of these complex responses. Force microscopy approaches provide a convoluted measure of passive and active adhesion components embedded in the integrin-based contacts.^13^ Atomic force microscopy (AFM) engages individual adhering cells to pull them vertically until detachment and retrieve a measure of the maximum normal adhesion force.^14,15^ In an alternative configuration, the cantilever is used to push or indent the cell, providing a local measure of its viscoelastic properties.^16,17^ Classic AFM measurements, however, suffer from poor scalability.^18^ Probe attachment and release from the cell are time-consuming, limiting the measurement capacity.^18,19^

The fluidic force microscopy (FluidFM)^20^ technology has significantly advanced the approach to single-cell force spectroscopy (SCFC)^14,15^, allowing the fast engagement and disengagement of probed cells.^18,21,22^ Thanks to the microfluidic control at the probe tip,^20^ serial quantitative measures can be performed with high temporal resolution. The improved measurement capacity of the system, together with its compatibility with standard live-cell microscopy, practically enables fast and serial cell population analysis^22^ in both the vertical pull out and pushing/poking configuration.^23^

Active forces exerted by cells on the substrate at the level of individual FAs, typically in the range of 10–30 nN, are captured by traction force microscopy (TFM). TFM uses fiducial landmarks embedded or engineered on a deformable substrate to track the displacement imposed by cell-generated contractility.^24−27^ From the resulting vectorial deformation field, the actuating forces can be inferred, based on the constitutive material model.^26,28−30^ An array of alternative protocols are available, aiming at optimizing the spatial resolution and force sensitivity of the method.^31^

TFM approaches require a step of reference image acquisition, which inherently affects the processability and the immediacy of force mapping.^32^ The reference-free confocal TFM (cTFM) technology bypasses this disruptive step, allowing for immediate rendering of displacement.^33^ A dedicated image processing algorithm, the Cellogram,^34^ completes the protocol enabling on-the-fly force map generation.^34^ The cTFM–Cellogram approach enables the time-resolved and multiplexed analysis of traction forces at the cell population level.

Here, we combine the FluidFM and cTFM–Cellogram technologies into a holistic force microscopy protocol, generating dynamic plots of basal forces obtained upon the apical cell manipulation with a microfluidic cantilever. The FluidFM and the cTFM were installed in direct contact with the apical and basal cell membrane, respectively (Figure 1). In particular, HeLa cells were selected for these experiments due to their well-established adhesion behavior^21,22,35^ and the possibility to manipulate adhesion and force generation by means of biochemical inhibitors.^36−38^

**Figure 1:**
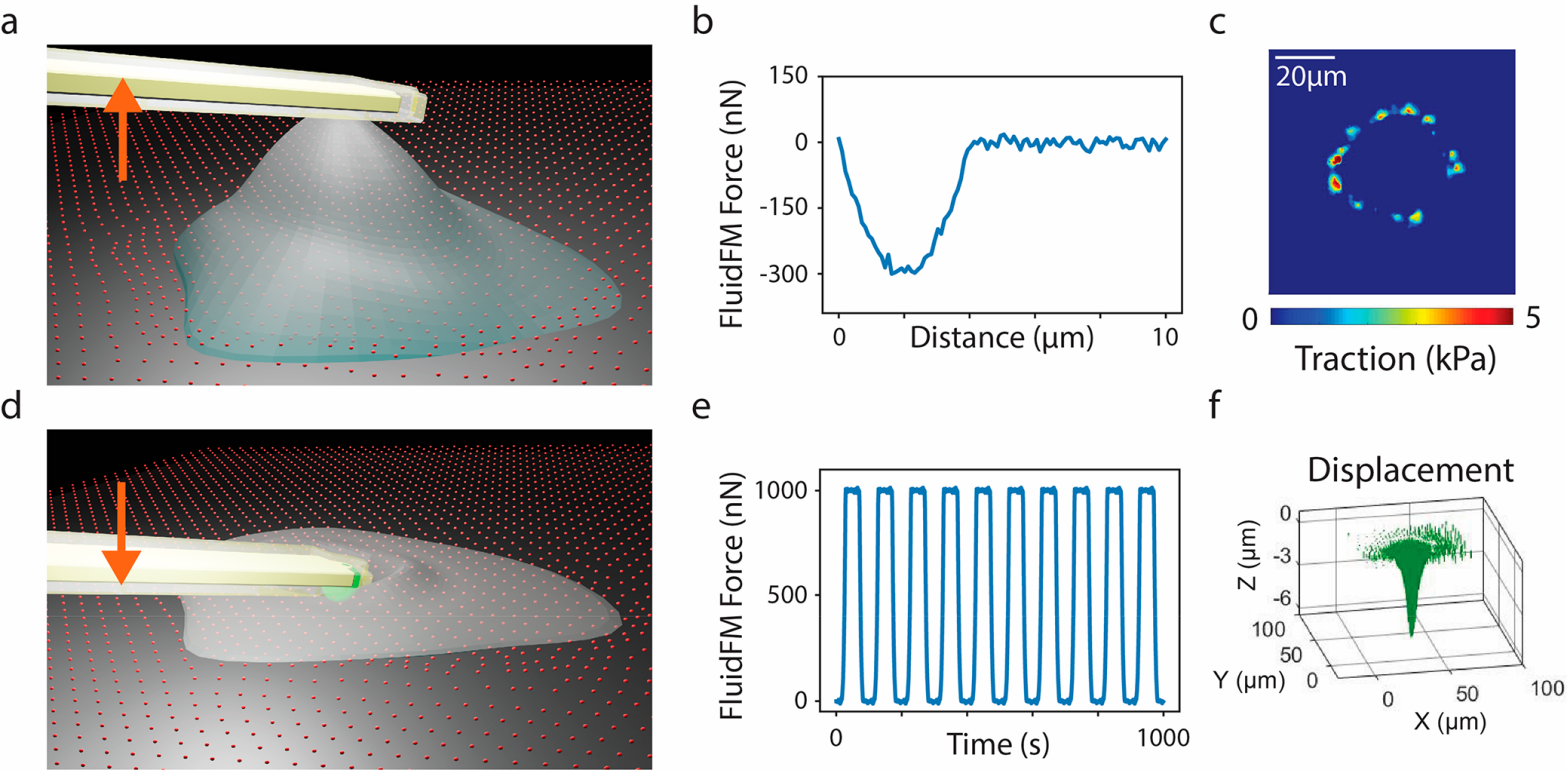
Schematic representation of the experimental set up. Cells seeded on cTFM samples and mounted on a FluidFM set up. (a) Cell detachment and synchronized measurement of maximum adhesion force and traction generation by FluidFM-based single-cell force spectroscopy. Tipless probes with an embedded microchannel, featuring an 8 μm circular aperture and spring constant of 4 N/m were used. Example of readout of iSCFS experiments from (b) FluidFM and (c) TFM. (d) Cyclic cell compression with a constant force of 1 μN and synchronized apical and basal measurements of force and traction generation. Tipless probes with an embedded microchannel, featuring a 2 μm circular aperture and spring constant of 2–3 N/m with a 6 μm bead mounted were used. Example of readout of poking experiments from (e) FluidFM and (d) TFM.

Integrated single-cell force spectroscopy (iSCFS) measurements were performed upon contacting the apical side of isolated cells with a tipless FluidFM probe (Figure 1). Cells were pulled vertically until detachment from the cTFM substrate, yielding a time-resolved force spectroscopy curve and a precise measurement of maximal adhesion force (Figure 1a,b). The concomitant displacement of the QD nanodisc array at the basal side, was recorded by fluorescent imaging. The map of actuating tractions was rendered using the Cellogram (Figure 1c).^34^ Typically, ∼5 min were required for the detachment of one individual cell, from the initial selection to the final disengagement. The steps of cell engagement, detachment, and disengagement leveraged the intrinsic advantages bestowed by the FluidFM technology.^21,22^ The software Cellogram ran in parallel without adding further delay to the process.^34^

Serial vertical poking experiments were achieved applying a vertical pushing force of 1 μΝ along the apico-basal axis of the cell (Figure 1d,e). Transmission to the basal side was simultaneously recorded through cTFM/Cellogram (Figure 1f). The readout consisted of a synchronized set of spatial–temporal force curves, obtained via the FluidFM at the apical side, and traction force maps rendered by the cTFM substrate and Cellogram analysis at the basal side (Figure 1). The duration of the full procedure depended on the number of compressive cycles. The time to complete one cycle was limited by the acquisition of multiple fluorescent images capturing the 3D displacement of the QD nanodisc array. The high quantum yield of QD nanodiscs (0.9^39^) allowed for fast image acquisition (25–100 ms), yielding complete Z-stacks in ∼20 s.

The maximum normal adhesion force of individual cells fluctuates significantly during the cell cycle,^21^ increases with senescence,^40^ and decreases with the transformation to a cancerous phenotype.^41^ These considerations imply that population measurements are required to obtain a reliable average. This is practically enabled by the FluidFM,^20,22^ which greatly (60 times, from hours to seconds) accelerates the engagement/disengagement phases of probing.^18^

Isolated HeLa cells interacting with cTFM substrates provided a viable substrate for FluidFM measurements (Figure 2a). Both the QD nanodisc arrays and the cells interacting with them were visualized by fluorescent and bright field microscopy, respectively. These images were used to obtain a spatially resolved map of QD nanodisc displacement and a measurement of the cell area and perimeter. Substrate displacement was computed in seconds and converted into a traction map via Cellogram within few minutes.^34^ Integrating the tractions over the entire cell surface yielded the corresponding measure of traction force (nN) exerted by the cell. The raw traction force values obtained for control HeLa cells were consistent with previous reports.^35^

**Figure 2:**
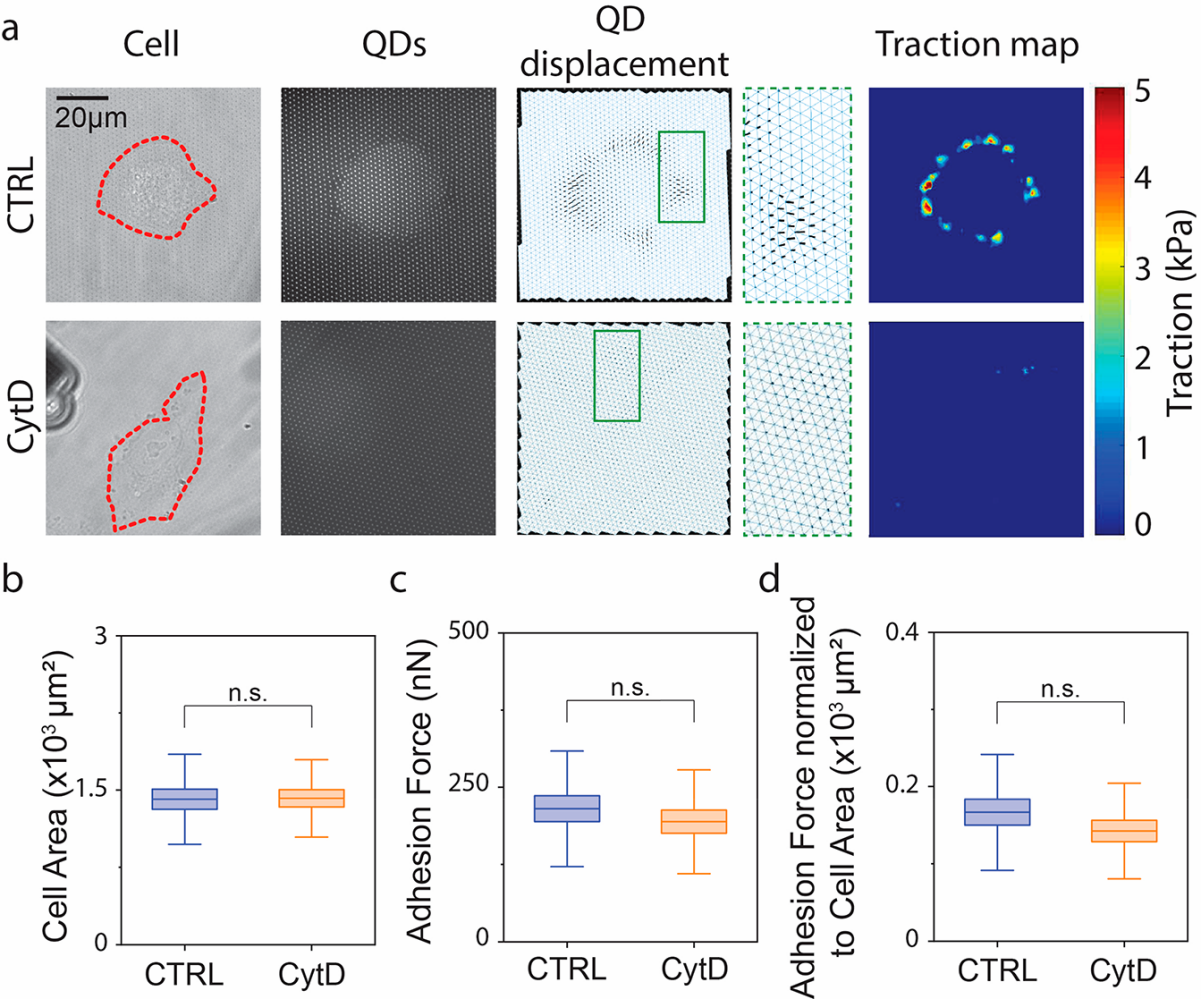
Mechanical response of HeLa cells to CytD. (a) Representative bright field (BF) images of individual control (CTRL) and CytD-treated (CytD) HeLa cells, the cell profile is highlighted by a red dashed line (left column; Cell), corresponding images of QDs (middle left column, QDs), displacement of the QDs (middle column; QD displacement) and magnification of deformed area (middle right column), corresponding traction map (right column; Traction map). Quantification of (b) cell area, (c) adhesion force, and (d) adhesion force normalized to cell area in control HeLa cells (CTRL) and upon CytD treatment (CytD) (*n*_CTRL_ = 20, *n*_CytD_ = 20, *n*′ ≥ 3). Boxplots indicate mean ± SEM, the population means are reported as a horizontal line inside the boxplot, whiskers report SD, n.s. stands for nonsignificant.

The actomyosin contractility of HeLa cells is efficiently blocked by cytochalasin D (CytD; SI Figure 1).^38,42^ CytD induced a clear reduction of substrate tractions (Figure 2a), whereas the overall cell area remained comparable to the control, indicating preserved adhesion (Figure 2b). FluidFM assessment of maximum adhesion force revealed a nonsignificant decrease of resistance to the vertical pull (Figure 2c), which remained negligible upon normalization to the cell area (Figure 2d). Separate testing with cTFM or FluidFM suggests that actomyosin generated tractions do not contribute to resisting vertical detachment.

iSCFS yields correlated FluidFM and traction force curves (Figure 3). Immediately after cantilever engagement (*t* = 0; FluidFM force > 0 nN), HeLa cells exerted tractions in the range of 400 nN (point A, 391.7 nN; Figure 3a). Upon actuation of the FluidFM, several phases could be distinguished based on the relative trend of the two force curves. In the initial phase (Phase I; yellow, Figure 3a), starting immediately after the onset of the vertical pull (FluidFM force < 0), both forces grew in intensity. The traction force increased by several nN (point B, 426.0 nN; Figure 3a) while the FluidFM force curve steadily moved to more negative values (0 s < *t* < 29 s). A second phase (Phase II; green, Figure 3a) started when the adhesion/pulling force reached its negative peak (*t* = 29 s; −270 nN; Figure 3a) and decreased thereafter. In this phase, the traction force decreased exponentially (point C, 124.8 nN; 29 s < *t* < 60 s). In the final phase (Phase III; blue, Figure 3a), the two curves reached a plateau. Interestingly, while the FluidFM force returned to 0, indicating full basal detachment of the engaged cells, residual foci of deformation were detected by the cTFM, resulting in delayed return to basal traction levels. Since the response of the elastomer in this range of deformations is fully elastic,^43^ this observation implies the presence of active adhesion components still interacting with the substrate.

**Figure 3:**
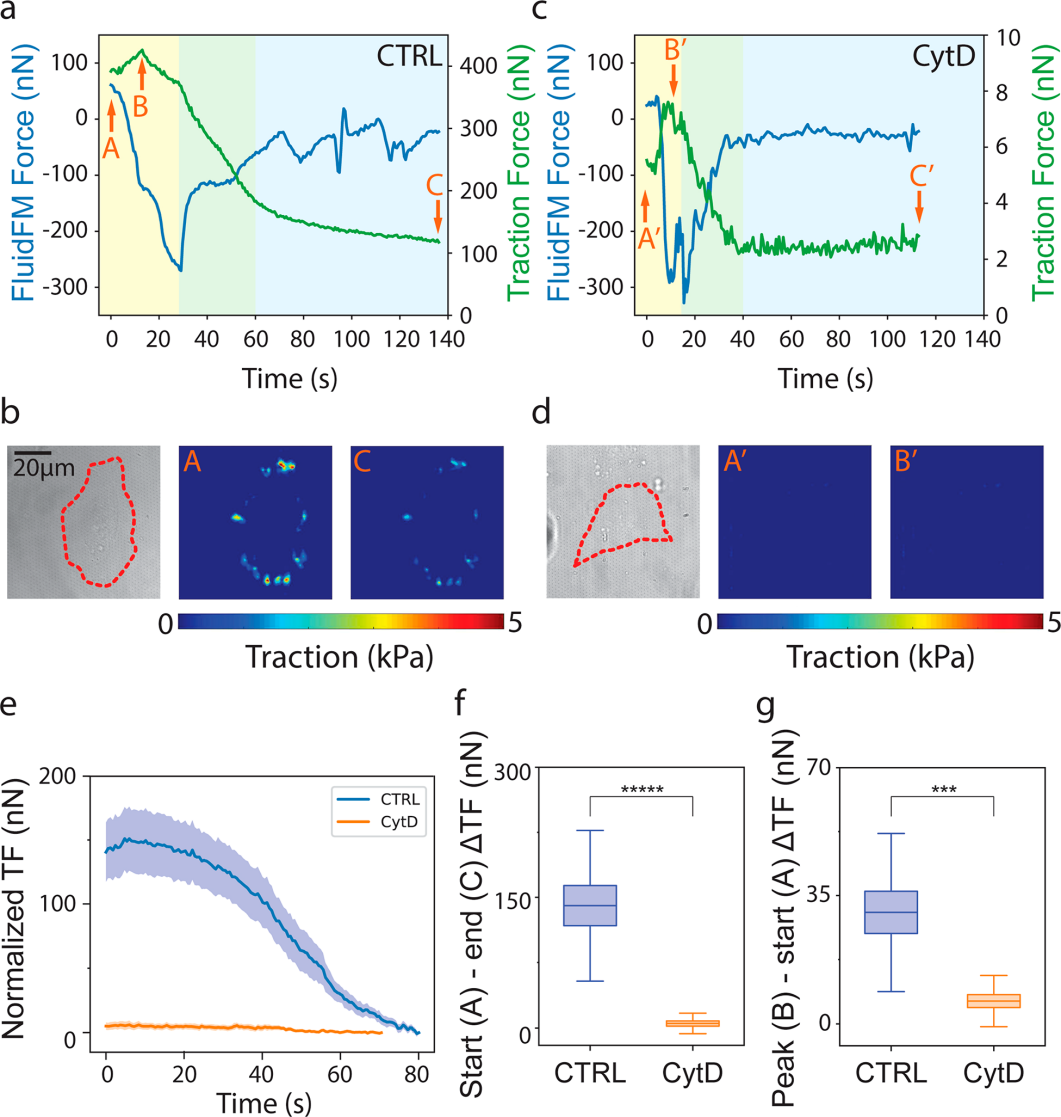
Time correlation of FluidFM force and generated traction in cell detachment. Representative FluidFM force curve (blue) and generated traction (green) over time during cell detachment of (a) control (CTRL) and (c) CytD-treated (CytD) HeLa cells. Phases I, II, and III are indicated with the background color of yellow, green, and blue, respectively. Positions A and A′, B and B′, and C and C′ indicate the starting point, the peak value, and the ending point of traction force curves, respectively. Corresponding bright field (BF) image of the (b) control and (d) CytD-treated HeLa cell. The cell profile is highlighted by a red dashed line (left panel). Traction force map in time points (b) A and C as show in panel a and (d) A′ and B′ as shown in panel c. (e) Quantification of normalized traction force curves in control (CTRL) and CytD-treated (CytD) HeLa cells. (f) Quantification of traction force change between the starting point (A/A′) and the ending point (C/C′) of the detachment in control (CTRL) and CytD-treated (CytD) HeLa cells. (g) Quantification of traction force change between the peak traction force value (B/B′) and the starting point (A/A′) of the detachment in control (CTRL) and CytD-treated (CytD) HeLa cells (*n*_CTRL_ = 14, *n*_CytD_ = 16, *n*′ ≥ 3). Line graphs and boxplots indicate mean ± SEM; in boxplots the population means are reported as a horizontal line inside the boxplot, and whiskers report SD, *** *p* < 0.001, ***** *p* < 0.00001.

In CytD-treated cells, the three phases described for control conditions were still evident but with different dynamics (Figure 3c,d). Phase I (0 s < *t* < 18 s; yellow, Figure 3c) led to a small but detectable increase in the basal deformation which was read as a peak of cTFM traction (point A′, 5.5 nN; point B′, 7.6 nN). Phase II proceeded faster than in the control (18 s < *t* < 40 s; green, Figure 3c) and led to a plateau close to 0 for both curves (Phase III; blue, point C′, 2.83 nN, Figure 3c). In this case, no residual signs of contractility were detected after cell detachment. Additional examples and supplementary control experiments with DMSO are shown in SI Figure 2 and SI Figure 3, respectively.

The difference in traction forces measured at the beginning of Phase I and at the end of Phase III, (Figure 3f) confirmed that treatment with CytD fully ablated cellular tractions (Figure 3e). On the other hand, the difference between the traction force measured at the peak (beginning of Phase II) and at the start (beginning of Phase I), indicated an increase in traction induced by the vertical pulling before detachment occurred (Figure 3g). This value was also significantly decreased by the treatment with CytD. These results indicate that, contrary to what was suggested by decoupled measurements (Figure 2d), planar traction supports cell resistance to vertical detachment, adding to the passive adhesion established by integrin receptors.

Next, the set up was utilized for harmonic localized apical compression (HLAC; Figure 1d–f). Upon contact with the cell, a tipless FluidFM cantilever with a reversibly immobilized 6 μm diameter bead, was moved downward to exert a controlled compression. Twenty cycles of periodic compression applying a force of 1 μΝ and full release were performed at a frequency of 0.0159 Hz. First, 10 cycles were actuated on a cytoplasmic region of the membrane. A second series of 10 cycles was then exerted on the nuclear region of the same cell. A volumetric map of the corresponding substrate deformation was obtained by tracking the 3D displacement of QD nanodiscs under the entire cell (Figure 1f), which rendered a representation of the force distribution.

The probe indentation (∼3.5–4 μm) exceeded the bead radius (3 μm). In these settings, the contact area between the probe and the cell apical membrane is approximated to half of the bead surface (56.5 μm^2^).^44^ Therefore, the applied force of 1 μΝ resulted in an apical compression of ∼18 kPa. This value is in the range of physiological pressure reported for cancer cells.^45^ In the HLAC experiments, the cell area and traction force exerted by probed individual cells did not vary significantly before and after the compression cycles (SI Figure 4). Additionally, the full viability of the HLAC protocol was demonstrated by a live/dead assay (SI Figure 5).

Two quantitative parameters were extracted. First, the region of substrate deformation (*R*_SD_), defined as the basal area affected by deformation upon apical compression. This value was normalized to a corresponding control, i.e., the area of deformation measured in a nearby region devoid of cells, upon direct FluidFM compression of the substrate (Figure 4a). Therefore, *R*_SD_ > 1 indicates that the transmitted apical compression affects a relatively larger region of the basal surface. *R*_SD_ < 1 indicates the opposite phenomenon; the apical force is focused on a smaller region of the substrate.

**Figure 4:**
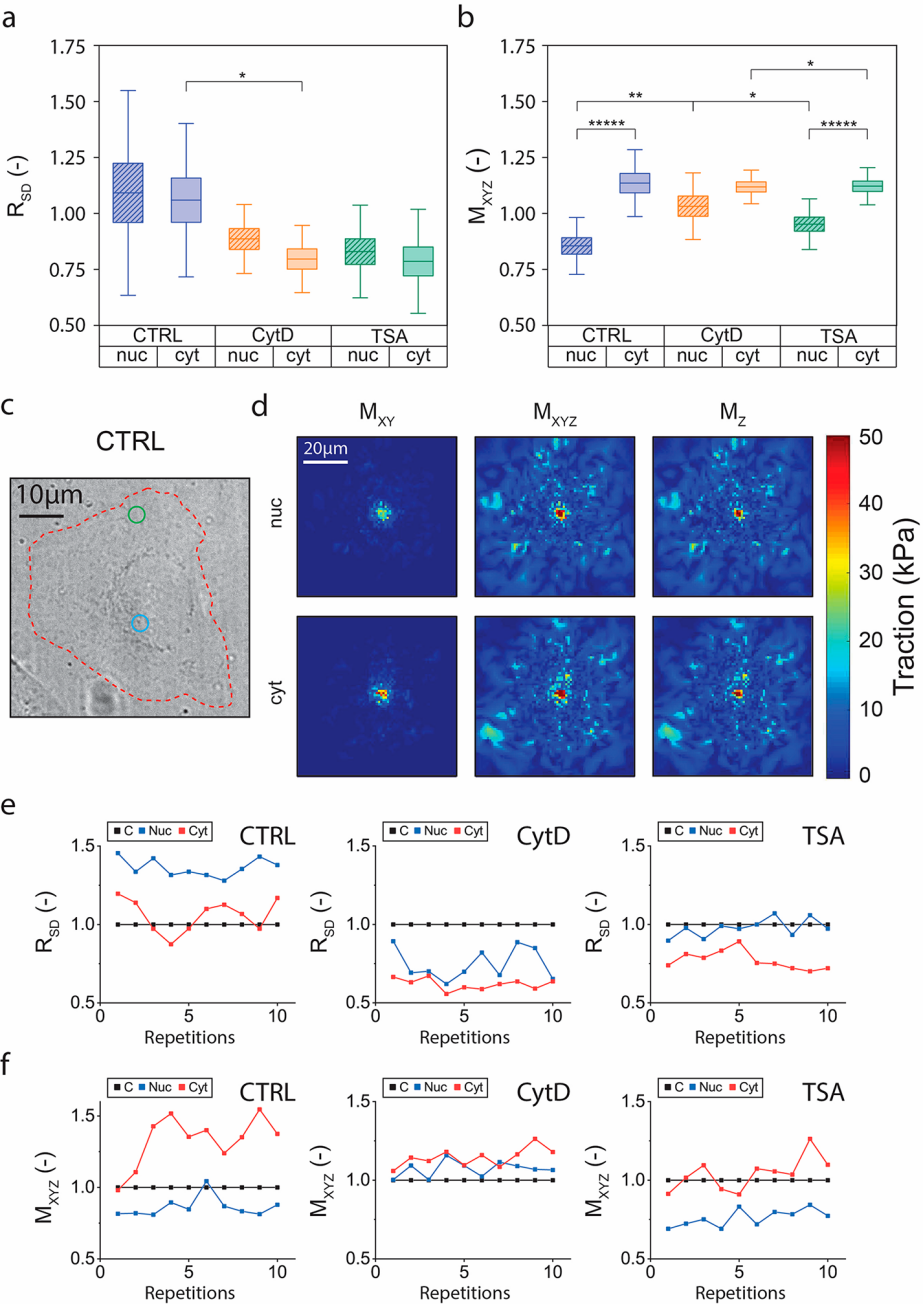
Poking on control and drug-treated HeLa cells. Quantification of (a) normalized affected area *R*_SD_ and (b) normalized traction *M*_*XYZ*_, during poking on the cell nucleus (nuc) and the cell cytoplasm (cyt) in control (CTRL), CytD-treated (CytD) and TSA-treated (TSA) HeLa cells (*n*_CTRL_ = 12, *n*_CytD_ = 11, *n*_TSA_ = 13, *n*′ ≥ 3). (c) Representative bright field image of a single HeLa cell before the experiment; cell profile is highlighted by a red dashed line, blue circle corresponds to poking position on the nucleus and green circle corresponds to poking position on the cytoplasm. (d) Representative traction maps of poking on the cell depicted in panel c. Full data of 10 poking cycles of (e) normalized affected area *R*_SD_ and (f) normalized traction *M*_*XYZ*_, during poking on control area without cells (C), the cell nucleus (nuc), and the cell cytoplasm (cyt) and in control (CTRL), CytD-treated (CytD) and TSA-treated (TSA) HeLa cells. Boxplots indicate mean ± SEM; the population means are reported as a horizontal line inside the boxplot, and whiskers report SD, * *p* < 0.05, ** *p* < 0.01, ***** *p* < 0.00001.

The second quantitative parameter is the magnitude of transmitted forces. The time-resolved variation of in-plane (*M*_*XY*_) and out-of-plane (*M*_Z_) forces was rendered during each phase of the periodic compression (Figure 4 and SI Figure 6). Additionally, the volumetric force magnitude (*M*_*XYZ*_) was computed and normalized to the control. *M*_*XYZ*_ > 1 indicates that upon stimulation the cell generates active forces adding to the compression, whereas *M*_*XYZ*_ < 1 implies that energy dissipation is taking place upon transmission of the actuating stimulus.

Control HeLa cells featured a mean value of *R*_SD_ > 1, during both cytoplasmic and nuclear probing (Figure 4a). In particular, 50% of cells showed values above 1 for almost all 10 subsequent compression cycles (SI Figure 7a). The other 50% showed values below 1 in response to at least 8 out of 10 compressions (SI Figure 7a). No evident temporal correlation for these fluctuations was found (Figure 4e). These results indicate that, on average, both the cytoplasmic and the nuclear region of HeLa cells transmitted the localized apical compression to an enlarged region of the basal substrate.

Interestingly, the evaluation of *M*_*XYZ*_ provided a clearly different trend. Localized compressions targeting the nuclear region of the cells under study, resulted in the corresponding transmission of a reduced force to the basal substrate compared to the control (*M*_*XYZ*_ = 0.85 ± 0.13; Figure 4b). The same stimulus was instead reinforced by the transmission of the applied force across the cell cytoplasm (*M*_*XYZ*_ = 1.14 ± 0.15; Figure 4b). This result suggests that the mechanical response of the two cellular compartments is opposite. While compression of the nucleus leads to energy dissipation or absorption by cellular components,^46,47^ the same stimulus induces the buildup of additional tractions when applied to the cytoplasm. A representative example of poking on a control single cell and the resulting *R*_SD_ and traction values, along with the corresponding traction maps are shown in Figure 4c–f.

To confirm this indication, we repeated the HLAC experiments upon biochemical inhibition of the cells’ actomyosin contractility by treating the cells with CytD. For these cells, the values of *R*_SD_ were typically below 1 (82% of cells and 84% of cycles; Figure 4a and SI Figure 7a). The major departure from the control was found in the *M*_*XYZ*_. In particular, while the cytoplasmic compressions yielded comparable values (*M*_*XYZ*_ = 1.12 ± 0.07; Figure 4b), the corresponding values after nuclear compressions were significantly increased (*M*_*XYZ*_ = 1.03 ± 0.15; Figure 4b), demonstrating that energy dissipation or absorption upon nuclear compression was not possible upon inhibition of actomyosin contractility.

To further evaluate the contribution of nuclear stiffness to this response, nuclear softening was artificially induced with trichostatin A (TSA), a histone deacetylase (HDAC) inhibitor that promotes chromatin relaxation.^48^ In TSA-treated cells, *R*_SD_ values were reduced below 1 for most cells (92% of cells and 89% of cycles; Figure 4a and SI Figure 7a), similarly to what was observed upon treatment with CytD. The nuclear and cytoplasmic *M*_*XYZ*_ were less changed and yielded values similar to the control (Figure 4b and SI Figure 7b).

The respective values for *M*_*Z*_ and *M*_*XY*_ for control and treated cells are presented in SI Figure 6 and SI Figure 7c,d. Additionally, representative examples of poking on single cells treated with CytD and TSA and the resulting *R*_SD_ and traction values, along with the corresponding traction maps are shown in Figure 4e,f and SI Figure 8. Supplementary control experiments with DMSO were performed to ensure the minimal effect of the vehicle (SI Figure 9). Altogether, these data indicate that nuclear stiffness contributes only minimally to the apico-basal transmission of compressive forces, whereas a major effect is dependent on cell-mediated contractility.

Cell cycle progression introduces changes to the mechanical fingerprint of mammalian cells, including variations in the mechanical stiffness and traction exerted.^35,38^ The Fucci sensor provides a direct tool to assess the cell cycle phase, based on a set of two fluorescent reporters, which are selectively activated upon cell cycle progression.^49^ Stably transfected HeLa cells (HeLa Fucci2)^50^ were used for the HLAC measurements presented in SI Figure 10 to restrict the measurements to cells in the same cell cycle phase. Specifically, cells in the S/G_2_ phase, which display a clear green fluorescent signal from the nucleus, were selected.

The results of cyclic compression on the S/G_2_ HeLa Fucci2 cells (SI Figure 10) reproduced the trend obtained in nonsynchronized HeLa cells (Figure 4 and SI Figure 6) for the response of both the cytoplasmic and nuclear regions. However, nuclear compressions rendered a lower value of *M*_*XYZ*_, indicating a reduced dissipation of compressive forces. This result is consistent with the concomitant reduction of the apparent Young’s modulus previously reported for the nuclei of HeLa cells in the S/G_2_ phases, which is related to the reorganization of the actin cytoskeleton.^38^

Finally, HLAC experiments were performed on primary human dermal fibroblasts (hFBs) to evaluate their response to apical compression. The experimental data (SI Figure 11) confirmed the tendency reported for HeLa cells, thus extending the conclusions to nontransformed human cells.

The establishment of substrate adhesions introduces asymmetry in the cell membrane, which goes beyond the assembly of localized anchoring points, the focal adhesions. Via the interaction with contractile cytoplasmic components of the cytoskeleton, it enables cell shape changes and the attainment of apico-basal polarity typical of epithelial tissues.^51^ The biochemical and molecular pathways governing these phenomena have been extensively described to reveal the complex mechanism regulating the interaction between the cell and its surroundings.^51,52^ At the adhesion points, the active generation of cellular forces leads to the remodeling of the extracellular environment and provides information to the cells in a process of bidirectional mechanical communication.^52^

Most of the extracellular physical stimuli passively experienced by adhering cells are delivered to their apical surface. This is the case for flow-generated wall shear stress on the luminal surface of endothelial cells^53^ and for cell induced deformations during the process of immune cell rolling over the endothelium.^5^ Similarly, apical shear paces interstitial cell advancement in a porous environment,^54^ and hydrostatic load controls the response of the inner linings in epithelial tissues.^55^ In all these examples, the transmission of forces across the cell, from the free surface to the one in contact with the substrate, is an integral part of the adaptive response.^56,57^

Atomic and traction force microscopy allow the manipulation and measurement of biological forces acting on and around adhering cells.^18,25,32,58,59^ The technological advancements leading to state-of-the-art FluidFM and cTFM–Cellogram protocols introduced sufficient measurement capacity to overcome intrinsic intercellular variability with population analysis.^21,22^

The two force measurement systems are developed to apply controlled mechanical stimuli to the free cell side (FluidFM) and read the cell-generated substrate deformation at the basal one (cTFM). The integration of the two technologies renders a holistic control of the mechanical environment juxtaposed to adhering cells, with high spatial and temporal resolution (Figure 1). To this end, the synchronization of apical stimulation and basal force readout introduces two major advancements. First, it provides time-resolved interactive basal feedback to the FluidFM readout of maximum normal adhesion force.^23^ Beyond the measurement of adhesion (Figure 2), iSCFS protocols, define various phases of cell detachment, which are instrumental to the comparison between control conditions and targeted biochemical treatments (Figure 3). The initial phase of vertical pulling is in fact actively counteracted by the cells, which increase contractility and therefore substrate deformation in the attempt to resist detachment (Figure 3). This process relies on an intact actin cytoskeleton and may be dysregulated in senescent cells, typically showing altered contractility and increased substrate adhesion.^40^

Second, the displacement of cTFM fiducial markers upon localized FluidFM apical compression, in the HLAC protocol, gives access to a detailed and time-resolved map of forces transmitted across the cell in response to a controlled apical stimulus (Figure 4). The convolution of passive force traveling across different cytoplasmic compartments and the overlapping active response provide indications of the potential mechanisms to dissipate mechanical insults. This is particularly relevant for the nuclear region, where compressions can induce membrane ruptures with loss of nuclear material in the cytoplasm.^60^ Experiments in which the active cell response is inhibited indicate that the intrinsic mechanisms reducing nuclear strain upon compression rely on a functional actin cytoskeleton, which could therefore act as a shock absorber. Within this framework, the mechanical properties of the nuclear region, defined by chromatin compaction, seem to play a lesser role. Nuclear compression is a key signal during metastatic cancer dissemination, inducing the expression of nuclear repair proteins, which confer resistance to chemotherapeutic drugs.^60,61^ This points to a link between dysregulated force generation and actin cytoskeleton response and the cell sensitivity to extracellular stimuli, with potential implications in cancer progression.

## Supporting Information Available

Detailed information about materials and methods. SI Figure 1, immunofluorescence images of HeLa cells. SI Figure 2, detachment of HeLa cells. SI Figure 3, effect of DMSO (vehicle control) in iSCFS measurements. SI Figure 4, evaluation before and after HLAC experiments. SI Figure 5, live/dead assay on HeLa cells after HLAC experiments. SI Figure 6, poking on control and drug-treated HeLa cells. SI Figure 7, quantification of percentage of poking cycles with value above 1. SI Figure 8, poking of individual control and drug-treated HeLa cells. SI Figure 9, effect of DMSO (vehicle control) in HLAC measurements. SI Figure 10, HLAC experiments on HeLa Fucci2 cells. SI Figure 11, HLAC experiments on human dermal fibroblast cells (hFBs). SI Figure 12, force–distance curve acquired by the FluidFM after poking on the substrate. SI Figure 13, determination of threshold for region of substrate deformation. (PDF)
Video S1, traction map of detachment of control HeLa cell (AVI)
Video S2, Traction map of detachment of CytD-treated HeLa cell (AVI)

## Supplementary Material

nl2c03733_si_001.pdf

nl2c03733_si_002.avi

nl2c03733_si_003.avi
