# Supplementary material for: 4D Force Detection of Cell Adhesion and Contractility
Source: Nano Lett. 2023 Mar 28;23(7):2467–75. doi: 10.1021/acs.nanolett.2c03733 (PMC10103301; doi:10.1021/acs.nanolett.2c03733)
Supplement: Supplementary file 1 — nl2c03733_si_001.pdf [file nl2c03733_si_001.pdf]

Supporting Information

4D force detection of cell adhesion and contractility

Nafsika Chala [a,ⱶ], Xinyu Zhang [b,ⱶ], Tomaso Zambelli [*,b], Ziyi Zhang [c], Teseo Schneider [d], Daniele Panozzo[*,c], Dimos Poulikakos [*,a], Aldo Ferrari [a,e,f]

a) Laboratory of Thermodynamics in Emerging Technologies, Department of Mechanical and Process Engineering, ETH Zurich, 8092 Zurich, Switzerland
b) Laboratory of Biosensors and Bioelectronics, Institute for Biomedical Engineering, ETH Zurich, 8092 Zurich, Switzerland
c) Courant Institute of Mathematical Sciences, New York University, New York, New York 10011, United States
d) Department of Computer Science, University of Victoria, Victoria, BC V8P 5C2, Canada
e) Experimental Continuum Mechanics, EMPA, Swiss Federal Laboratories for Material Science and Technologies, 8600 Dubendorf, Switzerland.
f) Institute for Mechanical Systems, Department of Mechanical and Process Engineering, ETH Zurich, 8092 Zurich, Switzerland

ⱶ these authors contributed equally

* email for Tomaso Zambelli: ztomaso@ethz.ch (FluidFM)

* email for Daniele Panozzo: panozzo@nyu.edu

* email for Dimos Poulikakos: dpoulikakos@ethz.ch

MATERIALS AND METHODS

Cell culture

HeLa cells were grown in DMEM medium (#41965039) supplemented with 10% v/v fetal bovine serum (FBS) and 1% v/v penicillin/streptomycin (P/S) (all reagents from ThermoFisher Scientific, USA) and were maintained at 37°C and 5% CO_2. HeLa Fucci2 cells stably expressing a Fucci construct [1] were purchased from Riken Bioresource Center (Japan) and previously characterized [2]. Primary adult human dermal fibroblasts were purchased from Cell Applications inc. (USA; #106-05a, lot #3092) and cultured in DMEM (#D6429, Sigma-Aldrich, USA) supplemented with 10% v/v FBS and 1% v/v P/S.

For the live experiments, CO_2 independent medium (#18045054, ThermoFisher Scientific, USA) supplemented with 10% v/v FBS and 1% v/v P/S was used.

Before the experiments, the substrates were coated with 10 µg/ml fibronectin (F1141, Sigma-Aldrich, USA) in PBS for at least 30 min at 37°C. The cells were seeded sparsely at a density of 1.2 x 10^3 cells/cm^2 on the substrate and let to fully spread overnight.

Drug treatments

In the experiments in which the actin cytoskeleton was perturbed, CO_2 independent medium with 600 nM cytochalasin D (C8273, Sigma-Aldrich, USA) was added for 30 min to the cells. Cells were then probed in the presence of cytochalasin D. For trichostatin A (T8552, Sigma-Aldrich, USA) treatment, cells were preincubated for 12-14 h with 200 ng/ml trichostatin A. Before the experiments, the medium was replaced with CO_2 independent medium. Additional control experiments were performed with the equivalent concentration of DMSO in CO_2 independent medium, serving as the vehicle control.

Live/Dead assay

To evaluate the viability of the cells after the HLAC experiments, a fluorescent live/dead assay (#L3324, ThermoFisher, USA) was used. After the HLAC experiments, the medium of the cells was replaced with the

live/dead solution which contained 4 nM of calcein-AM and 20 nM of ethidium homodimer-1 in culture medium. The cells were incubated for 20 min at 37°C and 5% CO_2 and then were imaged. Live cells were stained by green-fluorescent calcein-AM that indicates intracellular esterase activity, while dead cells were stained by red-fluorescent ethidium homodimer-1, which indicates loss of plasma membrane integrity.

Substrate fabrication

Glass coverslips were spin coated with CY52-276 silicone (DOWSIL, USA) with a mixing ratio of 9:10, which gives an elastic modulus of ~ 13 kPa [3-4]. Then, red-emitting quantum dot (QD) nanodiscs were deposited on the silicone substrates in monocrystalline arrays with a spacing of 2-3 µm using electrohydrodynamic nanodrip printing as previously described [3, 5]. The substrates were mounted on bottomless petri dishes for the experiments.

FluidFM

iSCFS experiments

Force-distance curves during detachment of single HeLa cells on cTFM substrates were measured by means of FluidFM-based single-cell force spectroscopy (SCFS). AFM-based (Nanosurf, Switzerland) FluidFM set up composed of a pressure controller (Cytosurge AG, Switzerland), a hollow microfluidic probe (Cytosuge AG, Switzerland) and an inverted microscope (Carl Zeiss, Germany) with a CMOS ORCA-Flash camera (Hamamatsu, Japan). The simultaneous recording of imaging information (shutter opening), deflection and piezo movement were integrated in a DAQmx device (BNC-2090A, National Instruments, US).

Optimized protocols were used to achieve complete detachment of single HeLa cells. Commercial tipless silicon nitride probes (Cytosurge AG, Switzerland) with an 8 µm circular aperture were chosen with high spring constants (4 N/m) for the detachment of cells within the 10 µm range of the AFM piezo. After thermal tuning calibration of the spring constants, the probes have a nominal value of 3.7 N/m. The deflection sensitivity was calibrated by averaging the slopes of deflection versus voltage of four forward

force spectra measured on glass in PBS (Thermofisher, USA) before each experiment. To prevent absorption of cells to the cantilever surface, cantilevers were coated with anti-fouling polymers. The cantilevers were first plasma-cleaned in air at 18 W for 30 s (Plasma Cleaner, Harrick Plasma, USA) and then coated with 2ml 0.1 mg/ml PMOXA (SusoS AG, Switzerland) in 1x HEPES for at least 4 h.

Experiments were performed at 37°C in an incubation chamber (Life Imaging Services, Switzerland) using the CO_2 independent medium. The cTFM samples were mounted on the live cell, widefield fluorescent microscope equipped with the FluidFM. Individual HeLa cells fully spread on the TFM arrays were selected. The approach set point of force spectra was set at 100 nN to ensure a good contact of the cantilever aperture and the cell membrane. Subsequently, a negative pressure of 100 mbar was applied during the 5 s pause time between the forward force spectra and backward fore spectra. Individual cells were detached by retracting the probe at 100 nm/s. The very slow speed of the detachment was selected to capture the cell contraction during the detachment process. Detached Hela cells were ejected away by an overpressure pulse.

HLAC experiments

To ensure the same contact area between the cantilever and the examined cells, a fluorescent polystyrene bead of 6 µm diameter (Degradex, USA) was fixed by a negative pressure of 800 mbar to the tipless silicon nitride probe (Cytosurge AG, Switzerland) with a 2 µm circular aperture [6]. The nominal spring constant of these cantilevers was 2.5 N/m after calibration. The deflection sensitivity was also carefully calibrated before indentation experiments. Moreover, we verified that the value of the applied force (i.e. measured with the FluidFM) in the control matched the value of the detected force (i.e. measured with the cTFM in the out-of-plane direction).

Ten repetitive force spectra were obtained with a 22 s pause time between forward and backward curve and between each repetition, allowing enough time for imaging acquisition, while holding the 1 µN applied force. Two poking experiments were realized on the same cell, first on the cell cytoplasm and then on the

cell nucleus. The position of each indentation was selected based on the bright field images of the HeLa cells.

The viability of the testing protocol was initially assessed, evaluating the cell area and the traction force exerted by individual HeLa cells before and after the end of the experimental procedure (SI Figure 4). Both values showed nonsignificant variations, indicating that the series of localized compressions on the cytoplasm and on the nucleus did not affect the cell adhesion or its ability to actively deform the substrate.

Substrate characterization

To confirm the elastic response of the cTFM substrate, in the range of deformation typically applied for the reported experiments, the force-distance curve obtained upon indentation with the FluidFM was evaluated. A bead with diameter of 10 µm (PS-FluoGreen-Fi226, microparticles GmbH, Germany) was fixed to the tipless silicon nitride probe (Cytosurge AG, Switzerland) with a circular aperture of 8 µm (3.7 N/m nominal spring constant) using a negative pressure of 800 mbar. The probe was calibrated according to established methods [7]. A force of 1.3 µN was then applied to the substrate with an indentation depth of approximately 6 µm. The resulting force-distance curve was obtained and analysed using linear regression. The success of linear fit (R^2 = 0.9943) indicates that the linearity of the substrate was still maintained at the indentation depth of 6 µm, and force of 1.3 µN (SI Figure 12).

Immunostaining

Cells were fixed with formaldehyde (#28908, Thermo Scientific, USA) in PBS, which was prewarmed at 37°C, for 15 min at RT and then washed with PBS. Then they were permeabilized with 0.5% Triton X-100 (#T8787, Sigma Aldrich, USA) in PBS for 10 min at RT. For the blocking of aspecific antibody binding, a solution of 5% bovine serum albumin (BSA) (#A6003, Sigma Aldrich, USA) in PBS for 1 h at RT was used. Subsequently, samples were incubated with primary antibodies at 4°C overnight. After rinsing three times with PBS, samples were incubated with secondary antibodies and the nuclear staining reagent DAPI for 45

min at RT. Finally, the samples were rinsed with PBS three times, and they were mounted with Fluoroshied (#F6182, Sigma Aldrich, USA).

Antibodies

The following commercial primary antibodies were used for immunofluorescence: mouse anti-paxillin (1:100, #610051, BD Bioscience, USA), rabbit anti-integrin β1 (1:100, #34971, Cell Signaling Technology, USA). The following secondary commercial antibodies were used: Alexa Fluor 488 donkey anti-mouse (1:200, #A21202, Invitrogen, USA) and Alexa Fluor 647 chicken anti-rabbit (1:200, #A21443, Invitrogen, USA). Actin cytoskeleton was stained with Phalloidin-TRITC (1:200, #P1951, Sigma Aldrich, USA). Cell nuclei were counterstained with DAPI (1:1000, #62248, Thermo Scientific, USA).

Imaging

The immunostained samples were imaged using an inverted Nikon-Ti spinning disk confocal microscope (Nikon, Japan) equipped with an Andor DU-888 camera (Oxford Instruments, UK) and a pE-100 LED illumination system (CoolLED Ltd, Andover, UK). Fluorescence images of immunostained HeLa cells were acquired with the 60×, 1.4 NA oil objective (Plan Apo, Nikon, Japan).

During the iSCFS experiments, 2D images of the QDs were recorded with an interval of 600 ms and 100 ms exposure time using the 63×, 1.3X NA oil objective (Carl Zeiss, Germany). During the HLAC experiments, a 3D Z-stack was acquired before and after every cycle with a Z-step of 0.3 µm and a 50 ms exposure time. Images were acquired using an inverted microscope (Carl Zeiss, Germany) equipped with a CMOS ORCA-Flash camera (Hamamatsu, Japan) and with an incubation chamber (Life Imaging Services, Switzerland) to control temperature and humidity.

TFM analysis

iSCFS experiments

For the calculation of the traction forces in the iSCFS experiments, the images of the QDs were analysed using the previously developed software Cellogram [8]. Without the need for a reference image, the theoretical initial position of the ordered array of the QDs and the images acquired during the experiments were used to define the displacement of the QD nanodiscs. Then, given the known material properties of the substrate, the tractions were computed. Finally, after integrating the tractions over the area, the traction forces were calculated.

A step of background removal was included in the analysis. An image of QDs devoid of cells close to the analysed cells was used as a background control. First, a filter of disk = 3 was applied and then all data was clamped with a threshold value. The threshold value was empirically selected for each sample so that the control image resulted to a traction force value close to zero.

HLAC experiments

For the calculation of the traction forces in the HLAC experiments, the 3D images of the QDs were analysed using an extended version of the software Cellogram. The extended version was built upon the algorithm proposed in [8], adapting it to detect 3D displacement of the QDs. The major algorithmic changes relate to the fact that the input was a 3D stack of confocal images instead of a single image. The markers in 3D were detected by fitting parametric cylindrical shapes for each marker using the primitive fitting algorithm introduced in [9]. To make the optimization problem continuous, the volumetric input dataset was approximated with a cubic B-spline. After the markers were detected, they were projected onto the petri dish plane to obtain the connectivity of the lattice, using the algorithm described in [8]. With the reconstructed lattice, the 3D displacements of the markers were used for the subsequent analysis. The analysis was performed on an adaptive tetrahedral mesh of the silicon substrate, using the Linear Elasticity material model with parameters E = 13.578 kPa and nu = 0.49. A reference implementation of the algorithm is available on the github repository of Cellogram:

https://github.com/cellogram/cellogram/tree/Cellogram2.

The region of substrate deformation (R_{SD}) was defined as the area of the substrate that had a vertical deformation larger than 1.75 µm. The threshold value for the vertical deformation was defined empirically. Different threshold values between 1 µm to 2.5 µm were evaluated as shown in SI Figure 13. The value of 1.75 µm was found to represent the smallest to experimentally provide a constant definition of R_{SD}, throughout the entire experiment and over all the compression cycles.

In-plane traction forces M_{XY} and out-of-plane traction forces M_Z, as well as M_{XYZ} were calculated for the R_{SD}. A single map of 3D traction forces was obtained at each cycle, upon the application of a compressive force of 1 µN. Specifically, the compression was maintained for the time necessary for the acquisition of a complete Z-stack (22 sec), rendering the displacement of QDs nanodiscs from the cTFM substate. Therefore, a single value of M was obtained for each compression cycle. All obtained values were normalized to the corresponding control value measured in a nearby region of the cTFM substrate devoid of cells, upon direct FluidFM compression of the substrate.

Data analysis

iSCFS experiments

A custom-made Labview program was used to record both TFM image acquisition and FluidFM output, enabling the time correlation of the two systems. For the generation of the consensus traction force plot (Figure 3e, SI Figure 3a), the data from individual experiments were aligned to the time point at which the FluidFM curve was zero after the cell approach, before the start of the iSCFS measurement. Subsequently, a normalization was performed so that the end point was set to zero. For experiments with a lower sampling rate, the 1-D function in Python was used to interpolate intermediate time points. In TFM-FluidFM plots (Figure 3a, c and SI Figure 2), the FluidFM force was demonstrated as a series of average values corresponding to the time points of the TFM image acquisition, due to the higher sampling rate of the FluidFM software, Nanosurf.

Image analysis

Cell area and perimeter

The area and the perimeter of HeLa cells was calculated with MATLAB (MathWorks, USA). Bright field images were used to manually draw the cell border. Cell profiles combined with the adopted magnification and pixel size of the microscope camera, were used to calculate the area and the perimeter of the cells.

Statistical analysis

The Shapiro-Wilk test was used to evaluate the normality of the data. For normally distributed data, the t-test was performed, while for nonnormally distributed data, the Mann–Whitney U test was performed. The χ^2-test was performed for categorical variables (above/below 1; SI Figure 7). For all tests, specificity of 0.05 was used. Bar graphs represent the total number of the population and line graphs depict the mean, while error bars visualize the standard error of the mean (SEM). In all box plots, the population means are reported as a horizontal line inside the boxplot, vertical length of the box represents the double of the SEM and whiskers report the double of the standard deviation (SD). The number of independent experiments is indicated as n', the number of total cells analyzed is shown as n and are reported in the figure legends.

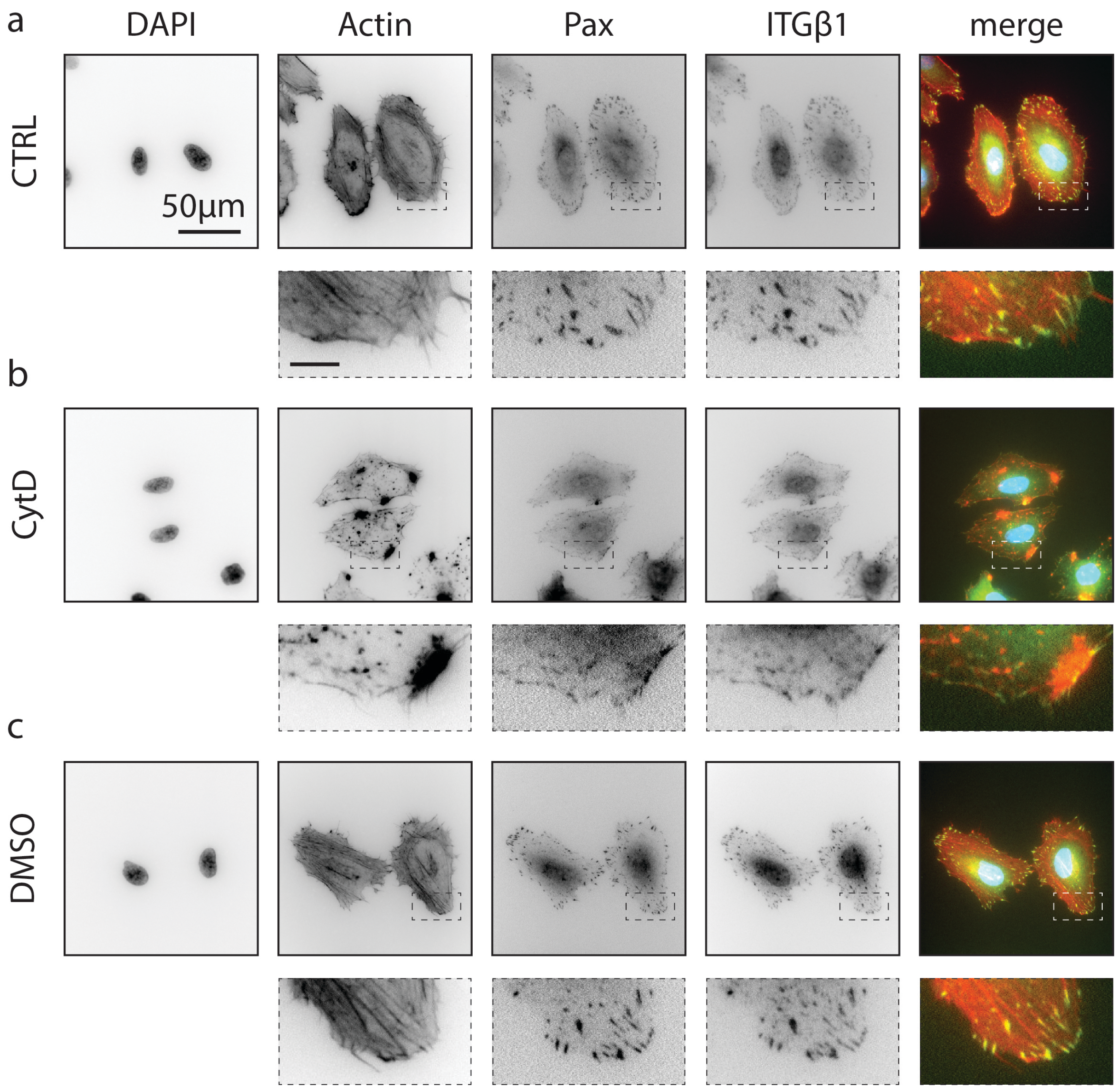

SI Figure 1. Immunofluorescence images of HeLa cells. Representative immunostaining of HeLa cells for nuclei (DAPI, inverted greyscale signal), actin cytoskeleton (Actin, inverted greyscale signal), paxillin (Pax, inverted greyscale signal) and integrin β1 (ITGβ1, inverted greyscale signal) in a) control cells (CTRL), b) cells treated with CytD for 1h (CytD) and c) cells treated with DMSO for 1h (DMSO). Corresponding magnified view of actin, paxillin and integrin β1 signal and overlapped signals of actin (red), paxillin (green) and nuclei (blue). Scale bar of magnified view is 10 μm.

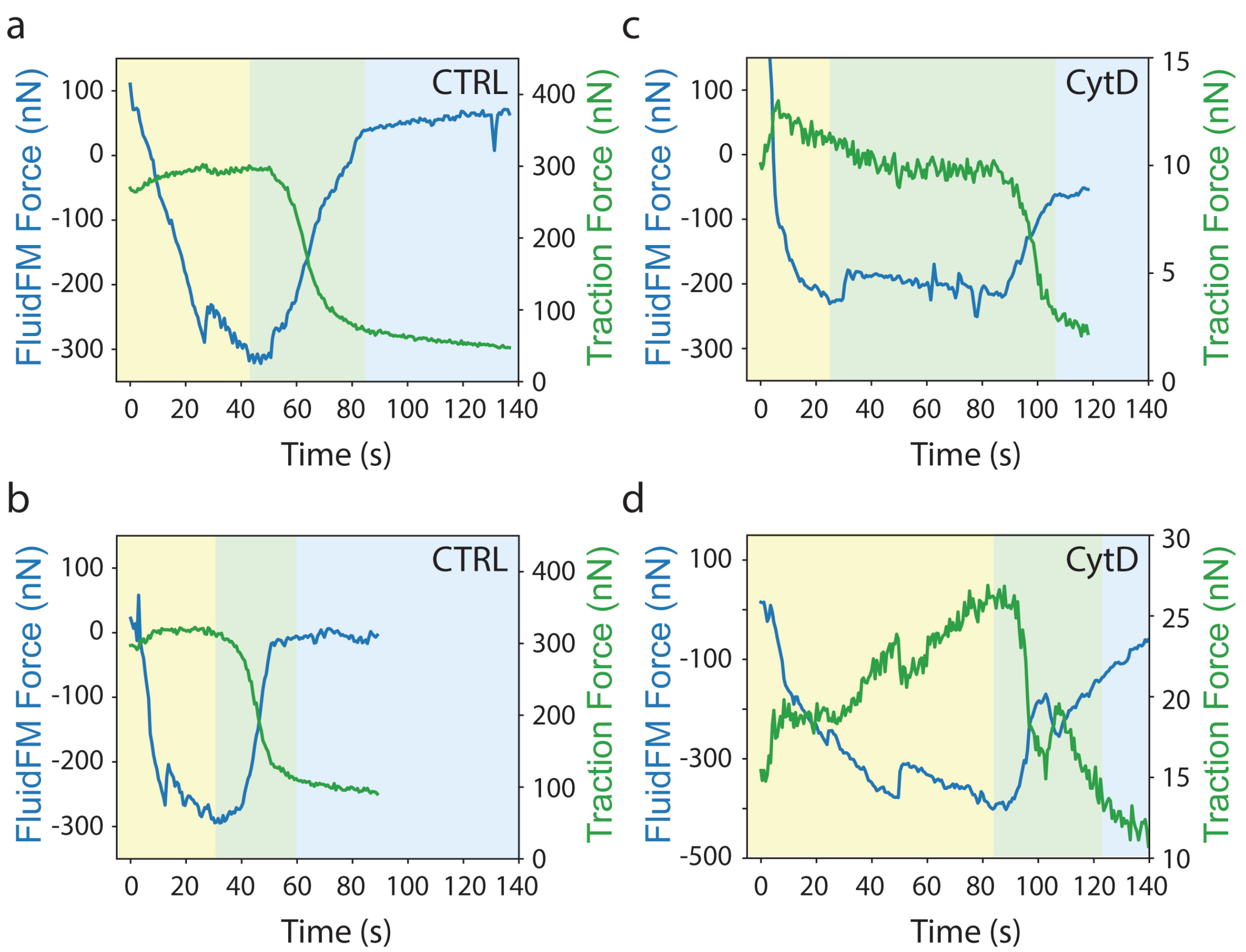

SI Figure 2. Detachment of HeLa cells. Representative FluidFM force curves (blue) and generated traction (green) over time during cell detachment of a,b) control (CTRL) and c,d) CytD-treated (CytD) HeLa cells. Phases I, II and III are indicated with the background color of yellow, green, and blue, respectively.

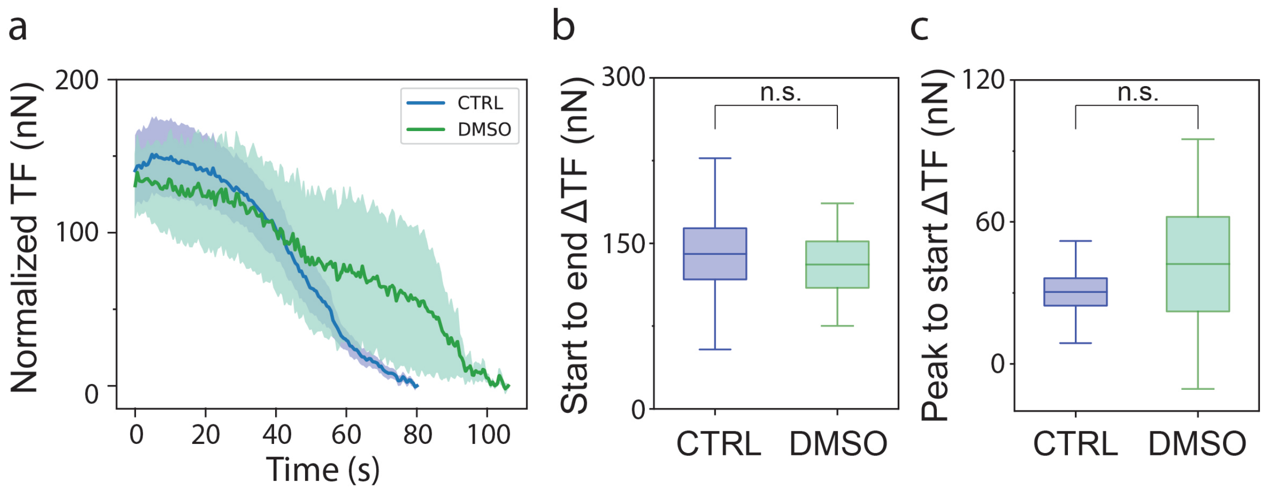

SI Figure 3. Effect of DMSO (vehicle control) in iSCFS measurements. a) Quantification of normalized traction force curves in (CTRL) and DMSO-treated (DMSO) HeLa cells. b) Quantification of traction force change between the start and the end of the detachment in (CTRL) and DMSO-treated (DMSO) HeLa cells. c) Quantification of traction force change between the peak traction force value and the start of the detachment in (CTRL) and DMSO-treated (DMSO) HeLa cells. ($n_{CTRL} = 14$, $n_{DMSO} = 7$, $n' \geq 3$). Line graphs and boxplots indicate mean ± SEM, in boxplots the population means are reported as a horizontal line inside the boxplot, whiskers report SD, n.s. stands for nonsignificant.

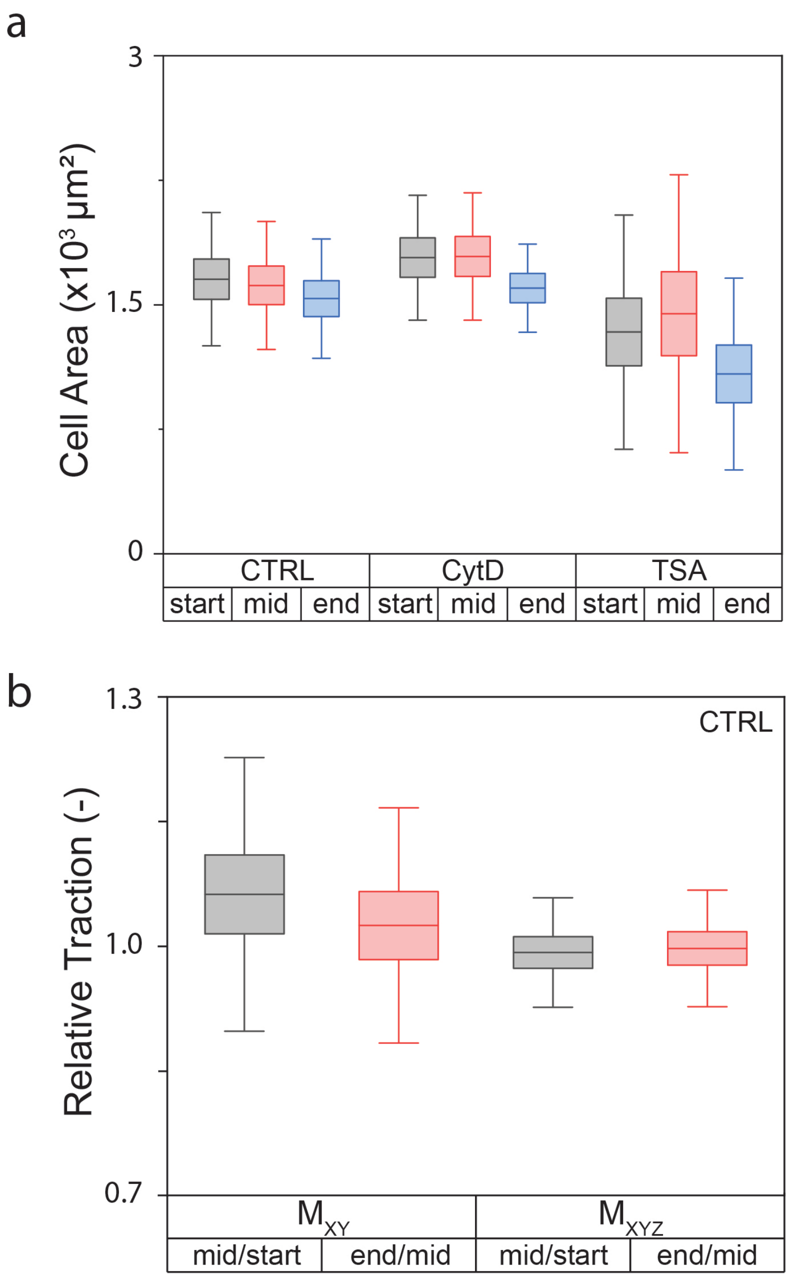

SI Figure 4. Evaluation before and after HLAC experiments. a) Quantification of the cell area before and after each poking experiment in control (CTRL), CytD-treated (CytD) and TSA-treated (TSA) HeLa cells. b) Quantification of the relative traction before and after each poking experiment in control (CTRL) HeLa cells. (n_{CTRL} = 11, n_{CytD} = 9, n_{TSA} = 9, n' ≥ 3). Boxplots indicate mean ± SEM, the population means are reported as a horizontal line inside the boxplot, whiskers report SD.

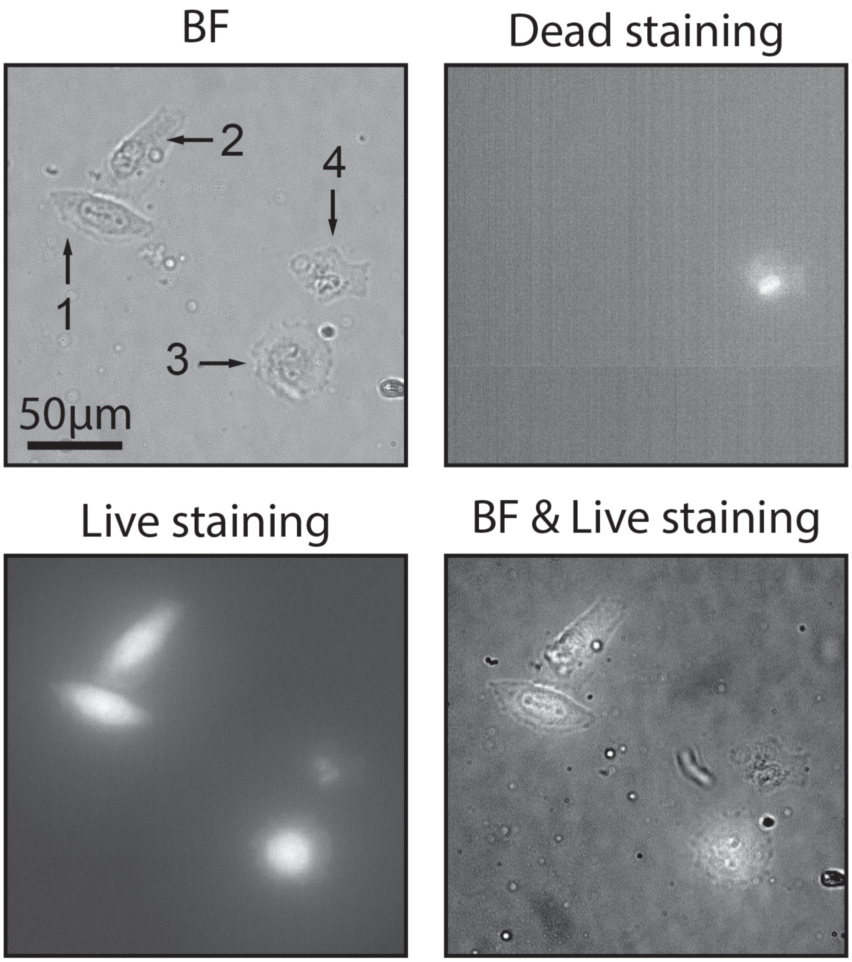

SI Figure 5. Live/dead assay on HeLa cells after harmonic localized apical compression. Bright field (BF) images and cell numbering (upper left), dead staining of ethidium homodimer-1 (upper right), live staining of calcein-AM (lower left) and overlay of BF and live staining of calcein-AM (lower right). Harmonic localized apical compression experiments were performed on cells #1, #2 and #3, while cell #4 was compressed with a higher force (>2 µN) and resulted in cell death. (n = 10, n' = 3).

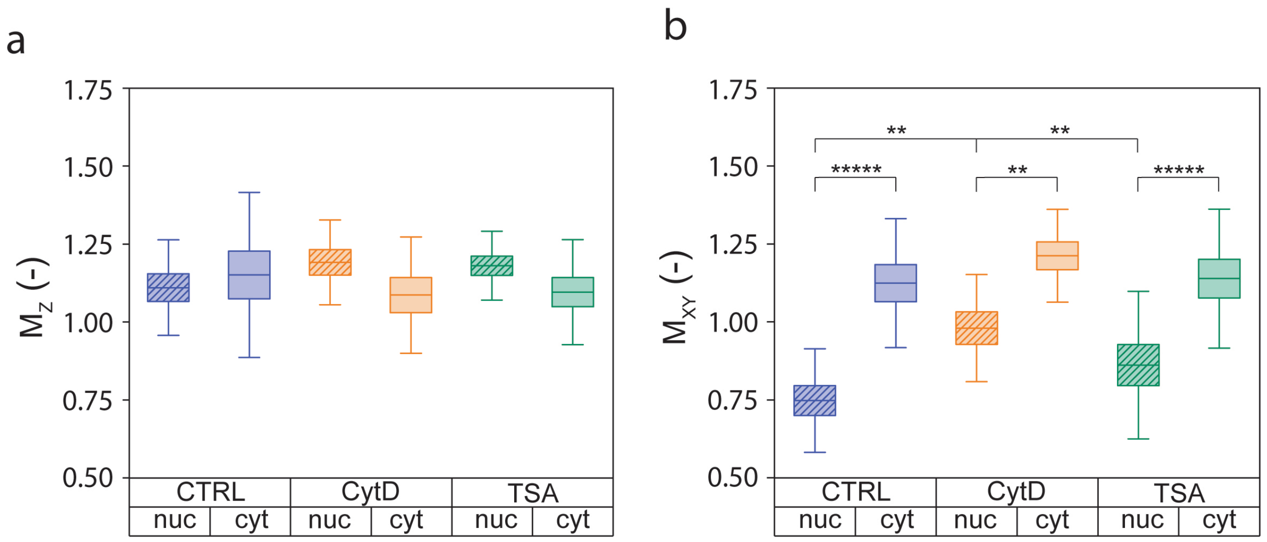

SI Figure 6. Poking on control and drug-treated HeLa cells. Quantification of a) normalized out-of-plane traction M_Z and b) normalized in-plane traction M_{XY}, during poking on the cell nucleus (nuc) and the cell cytoplasm (cyt) in control (CTRL), CytD-treated (CytD) and TSA-treated (TSA) HeLa cells. ($n_{CTRL} = 12$, $n_{CytD} = 11$, $n_{TSA} = 13$, $n' \geq 3$). Boxplots indicate mean ± SEM, the population means are reported as a horizontal line inside the boxplot, whiskers report SD, ** p<0.01, ***** p<0.00001.

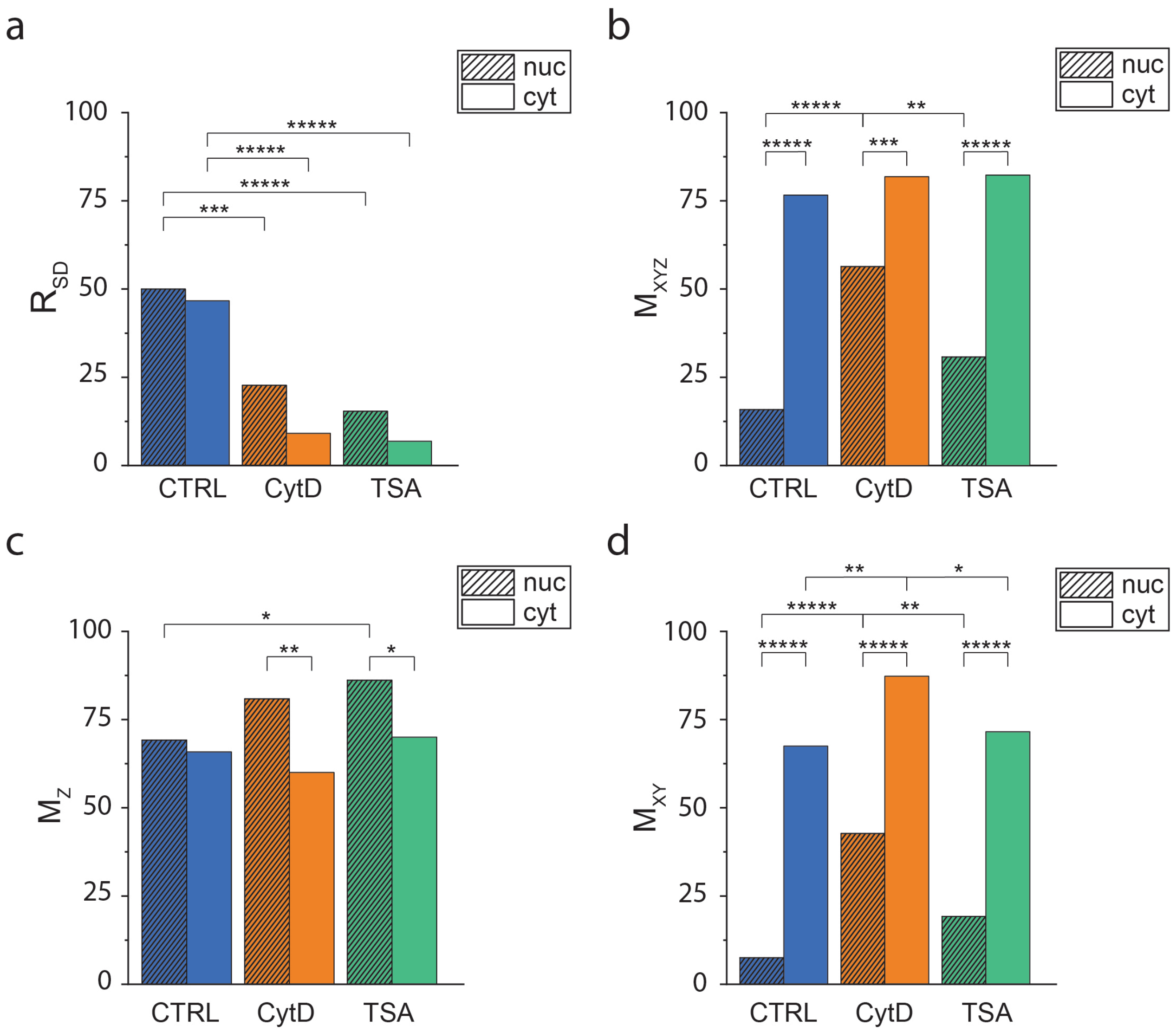

SI Figure 7. Quantification of percentage of poking cycles with value above 1 of a) normalized affected area R_{SD}, b) normalized traction M_{XYZ}, c) normalized out-of-plane traction M_Z and d) normalized in-plane traction M_{XY} of poking on the nucleus (nuc) and cytoplasm (cyt) of control (CTRL), CytD-treated (CytD) and TSA-treated (TSA) HeLa cells. ($n_{CTRL} = 120$, $n_{CytD} = 110$, $n_{TSA} = 130$, $n' \geq 3$) * $p<0.05$, ** $p<0.01$, *** $p<0.001$, ***** $p<0.00001$.

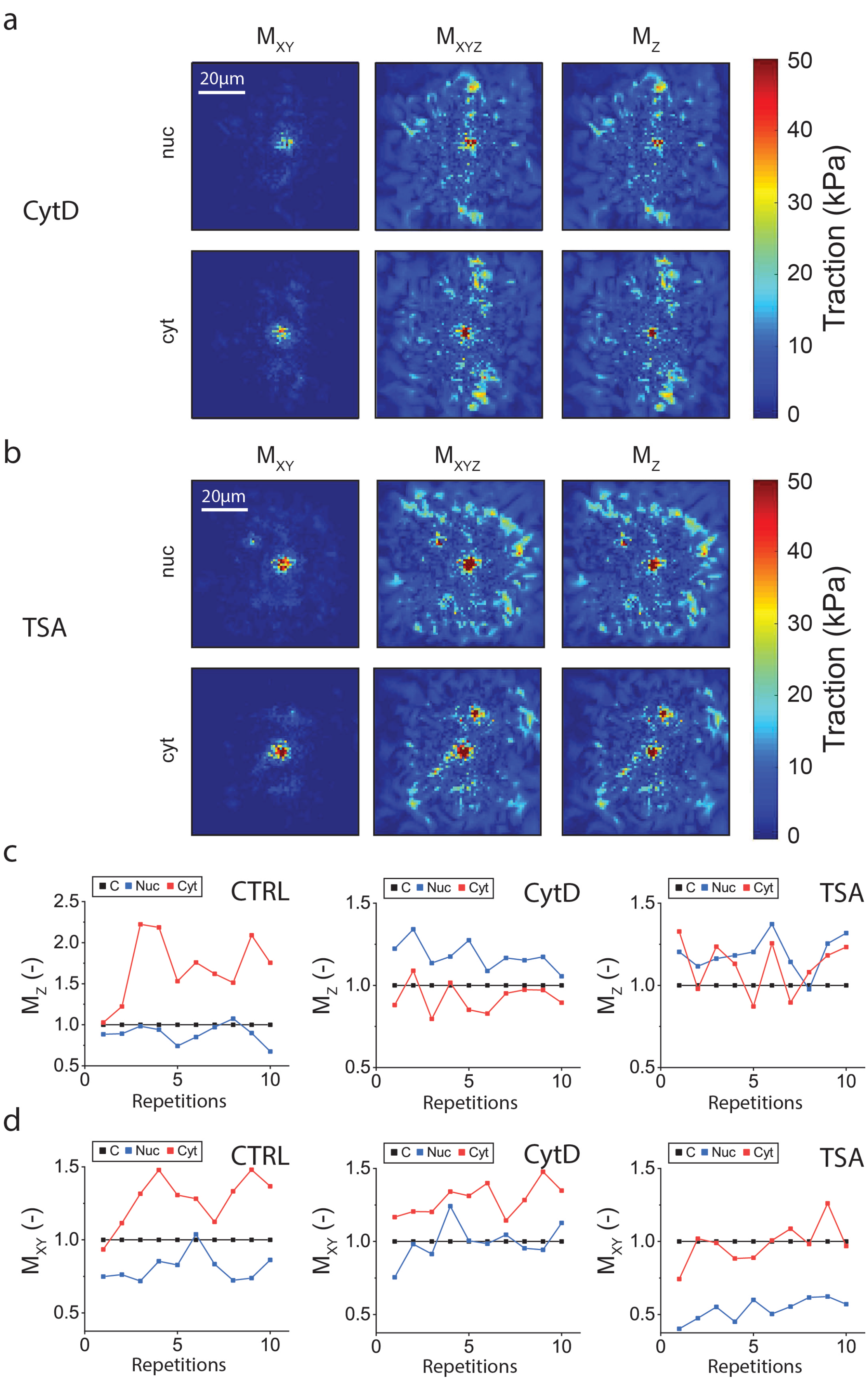
a
CytD
TSA
b
c
d
M_{XY}
M_{XYZ}
M_Z
nuc
cyt
20µm
Traction (kPa)
50
40
30
20
10
0
CTRL
CytD
TSA
C Nuc Cyt
M_Z (-)
M_{XY} (-)
Repetitions
2.5
2.0
1.5
1.0
0.5
0
5
10

SI Figure 8. Poking of individual control and drug-treated HeLa cells. Representative traction maps of poking on individual a) CytD-treated and b) TSA-treated HeLa cells. Full data of 10 poking cycles of c) normalized out-of-plane traction M_Z and d) normalized in-plane traction M_{XY}, during poking on control area without cells (C), the cell nucleus (nuc), the cell cytoplasm (cyt) in control (CTRL), CytD-treated (CytD) and TSA-treated (TSA) HeLa cells.

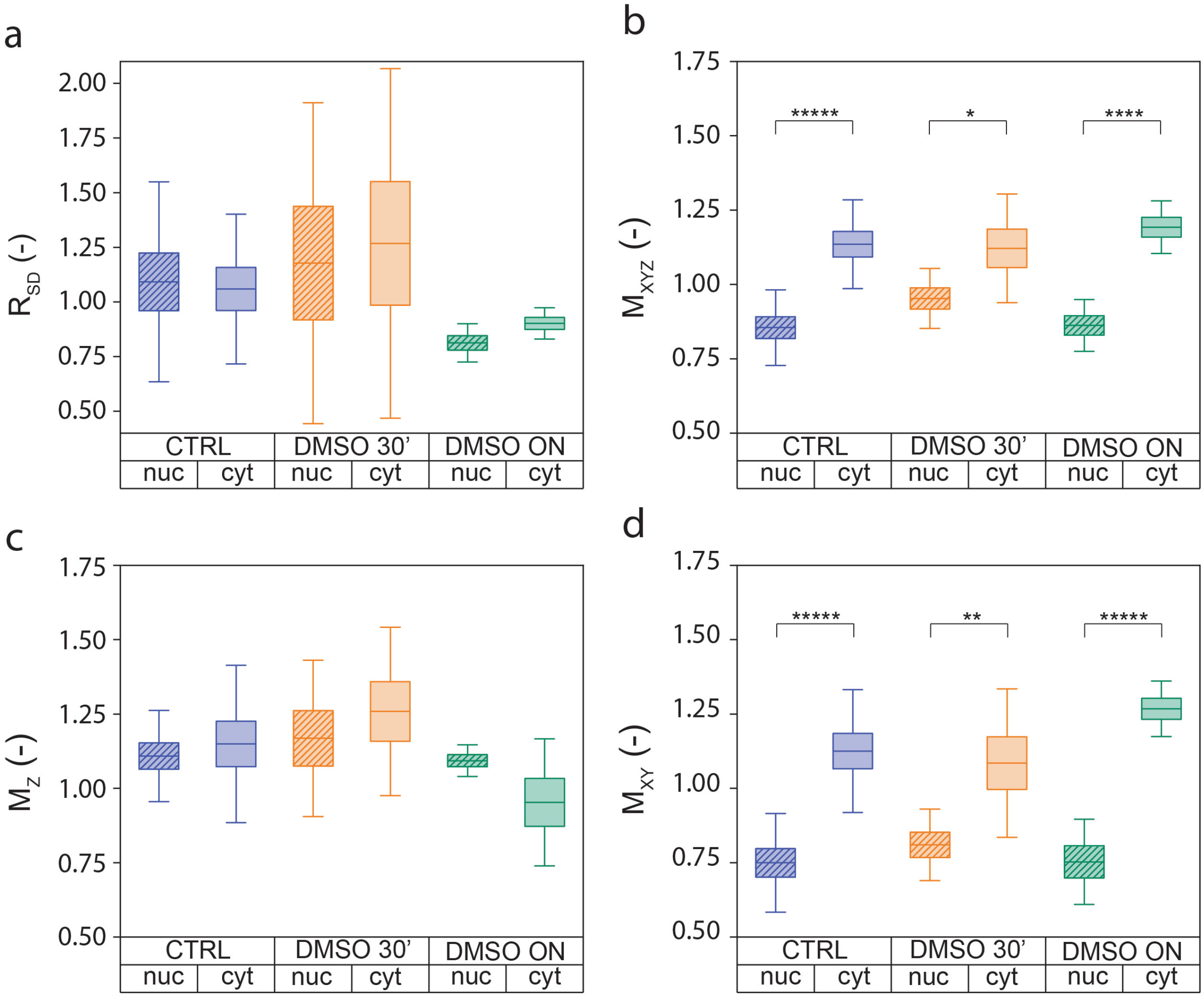

SI Figure 9. Effect of DMSO (vehicle control) in HLAC measurements. Quantification of a) normalized affected area R_{SD}, b) normalized traction M_{XYZ}, c) normalized out-of-plane traction M_Z and d) normalized in-plane traction M_{XY}, during poking on the cell nucleus (nuc) and the cell cytoplasm (cyt) in control (CTRL), DMSO-treated Hela cells for 30 min, similarly to the CytD treatment (DMSO 30’) and overnight for 12-14 h, similarity to the TSA treatment (DMSO ON). ($n_{CTRL} = 12$, $n_{DMSO\ 30'} = 8$, $n_{DMSO\ ON} = 7$, $n' \geq 3$) Boxplots indicate mean ± SEM, the population means are reported as a horizontal line inside the boxplot, whiskers report SD, * p<0.05, ** p<0.01, **** p<0.0001, ***** p<0.00001.

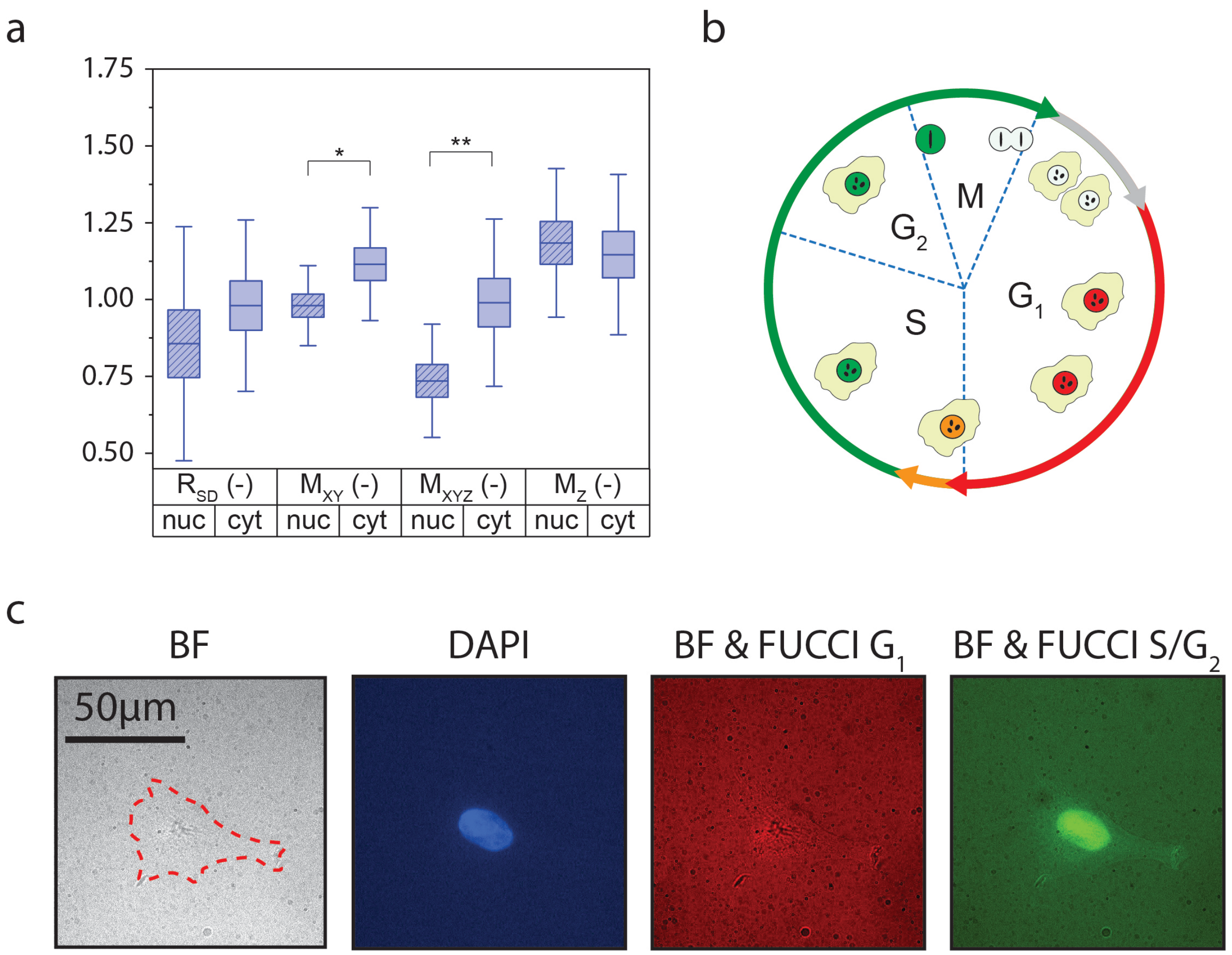

SI Figure 10. HLAC experiments on HeLa Fucci2 cells. a) Quantification of normalized affected area R_{SD}, normalized in-plane traction M_{XY}, normalized traction M_{XYZ} and normalized out-of-plane traction M_Z during poking on the cell nucleus (nuc) and the cell cytoplasm (cyt). (n = 12, n' = 4). b) Schematic of Fucci colours based on cell cycle phase. c) Representative images of a single HeLa Fucci2 cell in S/G_2, from left to right: bright field (BF), cell profile highlighted by a red dashed line, cell nuclei (DAPI), overlay of bright field and hCdt1 signal, a marker of cells in G_1 (BF & FUCCI G_1) and overlay of bright field and hGeminin, a marker of cells in S/G_2 (BF & FUCCI S/G_2).

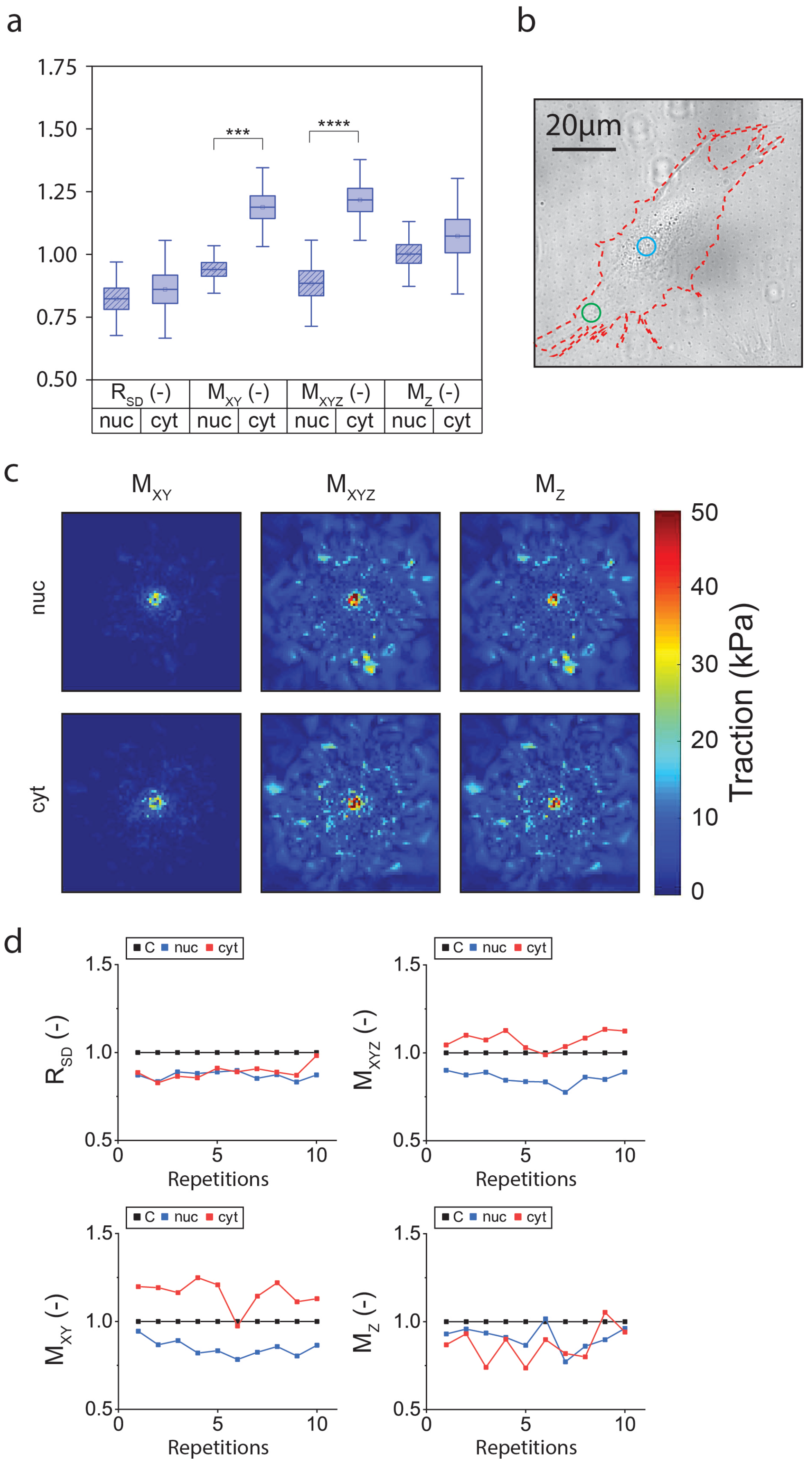
a
b
c
d
20µm
***
****
R_{SD} (-)
M_{XY} (-)
M_{XYZ} (-)
M_{Z} (-)
nuc
cyt
Traction (kPa)
C nuc cyt
Repetitions

SI Figure 11. HLAC experiments on human dermal fibroblast cells (hFBs). a) Quantification of normalized affected area R_{SD}, normalized in-plane traction M_{XY}, normalized traction M_{XYZ} and normalized out-of-plane traction M_Z during poking on the cell nucleus (nuc) and the cell cytoplasm (cyt). (n = 12, n' = 2). b) Representative bright field image of a single hFB cell before the experiment, cell profile highlighted by a red dashed line, blue circle corresponds to poking position on the nucleus and green circle corresponds to poking position on the cytoplasm. c) Representative traction maps of poking on the cell depicted in b). d) Full data of 10 poking cycles of normalized affected area R_{SD}, normalized traction M_{XYZ}, normalized in-plane traction M_{XY} and out-of-plane traction M_Z during poking on control area without cells (C), the cell nucleus (nuc) and the cell cytoplasm (cyt). Boxplots indicate mean ± SEM, the population means are reported as a horizontal line inside the boxplot, whiskers report SD, *** p<0.001, **** p<0.0001.

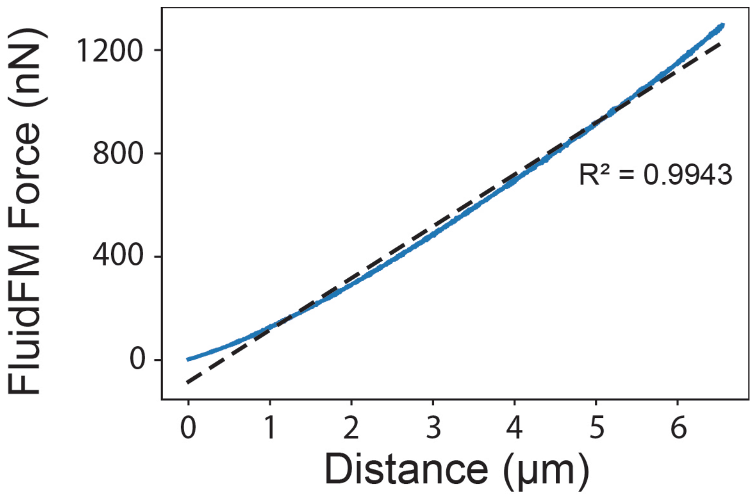

SI Figure 12. Force-distance curve acquired by the FluidFM after poking on the substrate.

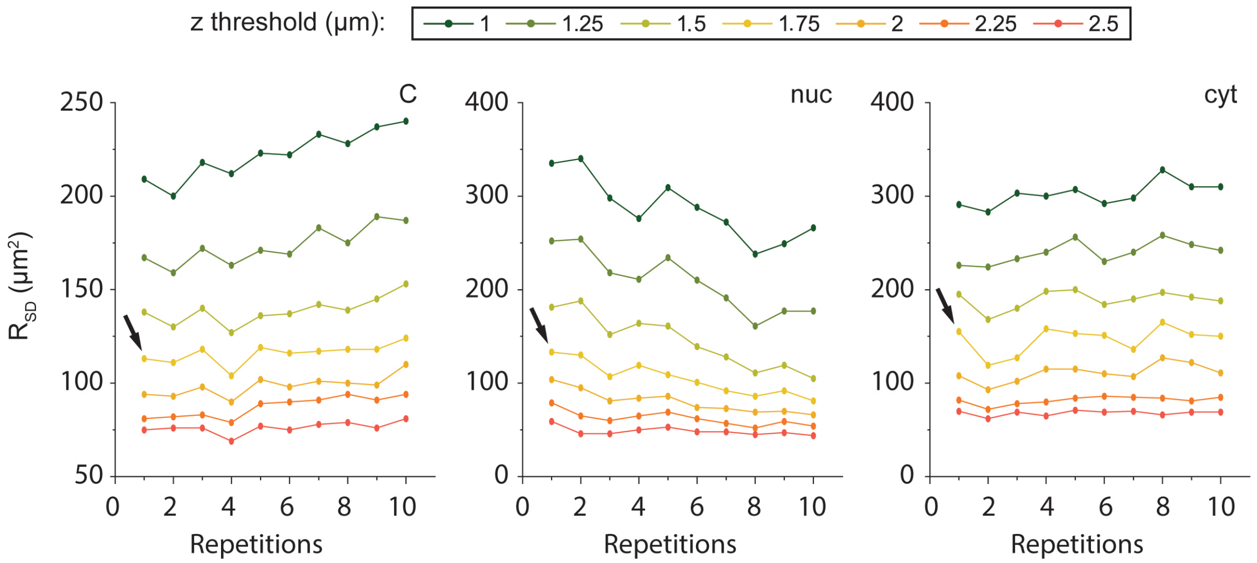

SI Figure 13. Determination of threshold for region of substrate deformation (R_{SD}). Region of substrate deformation after HLAC experiment on control area without cells (C), the cell nucleus (nuc) and the cell cytoplasm (cyt) and for different z thresholds values.

References

1. Sakaue-Sawano, A.; Kobayashi, T.; Ohtawa, K.; Miyawaki, A., Drug-induced cell cycle modulation leading to cell-cycle arrest, nuclear mis-segregation, or endoreplication. *BMC Cell Biol* **2011,** *12*, 2.
2. Panagiotakopoulou, M.; Bergert, M.; Taubenberger, A.; Guck, J.; Poulikakos, D.; Ferrari, A., A Nanoprinted Model of Interstitial Cancer Migration Reveals a Link between Cell Deformability and Proliferation. *ACS Nano* **2016,** *10* (7), 6437-48.
3. Bergert, M.; Lendenmann, T.; Zundel, M.; Ehret, A. E.; Panozzo, D.; Richner, P.; Kim, D. K.; Kress, S. J.; Norris, D. J.; Sorkine-Hornung, O.; Mazza, E.; Poulikakos, D.; Ferrari, A., Confocal reference free traction force microscopy. *Nat Commun* **2016,** *7*, 12814.
4. Reyes Lua, A. M.; Hopf, R.; Mazza, E., Factors influencing the mechanical properties of soft elastomer substrates for traction force microscopy. *Mechanics of Soft Materials* **2020,** *2* (1), 6.
5. Galliker, P.; Schneider, J.; Eghlidi, H.; Kress, S.; Sandoghdar, V.; Poulikakos, D., Direct printing of nanostructures by electrostatic autofocussing of ink nanodroplets. *Nat Commun* **2012,** *3*, 890.
6. Dorig, P.; Ossola, D.; Truong, A. M.; Graf, M.; Stauffer, F.; Voros, J.; Zambelli, T., Exchangeable colloidal AFM probes for the quantification of irreversible and long-term interactions. *Biophys J* **2013,** *105* (2), 463-72.
7. Sader, J. E.; Chon, J. W. M.; Mulvaney, P., Calibration of rectangular atomic force microscope cantilevers. *Rev Sci Instrum* **1999,** *70* (10), 3967-3969.
8. Lendenmann, T.; Schneider, T.; Dumas, J.; Tarini, M.; Giampietro, C.; Bajpai, A.; Chen, W.; Gerber, J.; Poulikakos, D.; Ferrari, A.; Panozzo, D., Cellogram: On-the-Fly Traction Force Microscopy. *Nano Lett* **2019,** *19* (10), 6742-6750.
9. Yamaguchi, N.; Zhang, Z.; Schneider, T.; Wang, B.; Panozzo, D.; Knaut, H., Rear traction forces drive adherent tissue migration in vivo. *Nat Cell Biol* **2022,** *24* (2), 194-204.
